# Fast live-cell conventional fluorophore nanoscopy with ImageJ through super-resolution radial fluctuations

**DOI:** 10.1038/ncomms12471

**Published:** 2016-08-12

**Authors:** Nils Gustafsson, Siân Culley, George Ashdown, Dylan M. Owen, Pedro Matos Pereira, Ricardo Henriques

**Affiliations:** 1 Quantitative Imaging and Nanobiophysics Group, MRC Laboratory for Molecular Cell Biology, Department of Cell and Developmental Biology, University College London, Gower Street, London WC1E 6BT, UK; 2 Centre for Mathematics and Physics in Life Sciences and Experimental Biology (CoMPLEX), University College London, Gower Street, London WC1E 6BT, UK; 3 Department of Physics, Randall Division of Cell and Molecular Biophysics, King's College London, London SE1 1UL, UK

## Abstract

Despite significant progress, high-speed live-cell super-resolution studies remain limited to specialized optical setups, generally requiring intense phototoxic illumination. Here, we describe a new analytical approach, super-resolution radial fluctuations (SRRF), provided as a fast graphics processing unit-enabled ImageJ plugin. In the most challenging data sets for super-resolution, such as those obtained in low-illumination live-cell imaging with GFP, we show that SRRF is generally capable of achieving resolutions better than 150 nm. Meanwhile, for data sets similar to those obtained in PALM or STORM imaging, SRRF achieves resolutions approaching those of standard single-molecule localization analysis. The broad applicability of SRRF and its performance at low signal-to-noise ratios allows super-resolution using modern widefield, confocal or TIRF microscopes with illumination orders of magnitude lower than methods such as PALM, STORM or STED. We demonstrate this by super-resolution live-cell imaging over timescales ranging from minutes to hours.

## Abstract (web summary)

Super-resolution fluorescent imaging typically makes use of intense phototoxic illumination. Here the authors achieve live-cell super-resolution imaging using low-illumination standard microscopes with the aid of a new analytical approach called Super-Resolution Radial Fluctuations (SRRF), provided as an ImageJ plugin.

## Graphical abstract

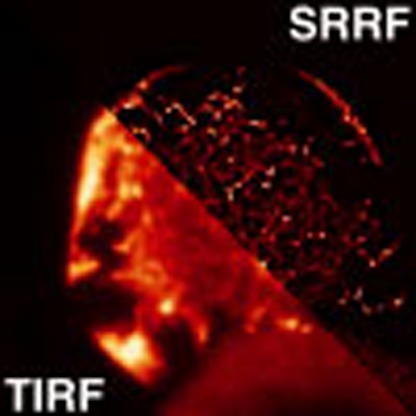

Camera based super-resolution approaches such as photoactivated localization microscopy (PALM)^1^ and Stochastic Optical Reconstruction Microscopy (STORM)^2^ have become well-established methods for structural cell biology studies, achieving lateral resolutions under 30 nm. Comparably, structured illumination microscopy (SIM)^3^, is an attractive alternative approach for live-cell super-resolution due to the reduced illumination requirements, fast acquisition rates and compatibility with conventional fluorophores such as green-fluorescent protein (GFP). SIM, however, requires expensive additional optical components to achieve resolutions on the order of ∼150 nm. As a consequence, recent years have seen considerable focus put on adapting PALM- and STORM-like approaches to allow live-cell nanoscopy. These single-molecule localization microscopy (SMLM) approaches circumvent Abbe's diffraction limit through the acquisition of a large sequence of frames (typically thousands), each containing a small population of transiently emitting non-overlapping fluorophores. The analytical detection and localization of these individually resolvable fluorophores populates a highly accurate map of fluorophore positions^1^,^2^.

Live-cell SMLM depends on the capacity to detect enough fluorophores to super-resolve structures, in a time small enough to minimize motion-blur artefacts^4^,^5^,^6^. A recent analysis of >30 software packages designed for processing SMLM data^7^ shows that these algorithms are capable of approaching the Cramér–Rao theoretical precision limit^8^ when the distance between emitting fluorophores is >∼1.5 μm. Some specialized ‘high-density' algorithms are capable of localizing fluorophores separated by distances in the range of 1.5 μm down to 0.15 μm at the cost of poorer precision^7^,^9^. Nonetheless, at smaller separations (or equivalently, higher densities), even these algorithms suffer from substantial imprecision, poor recall and artefacts^7^. As a consequence, it becomes critical to maintain the density of fluorophores actively emitting in each acquired frame within the boundaries of the analytical approach used. This task is difficult or impossible when dealing with dynamic processes in cells that frequently present a large heterogeneity in fluorophore densities. Three algorithms in particular have overcome this maximum density limit by circumventing the requirement for individual fluorophore localizations. These are deconSTORM^10^, which reconstructs super-resolution images by averaging extensively deconvolved images of sub-populations of fluorophores; 3B (ref. 11), which uses Bayesian analysis; and super-resolution optical fluctuation imaging (SOFI)^12^, which uses higher-order statistical analysis of temporal fluorophore intensity fluctuations.

Here, we present a novel analytical approach, super-resolution radial fluctuations (SRRF), which follows similar principles to deconSTORM, 3B and SOFI, where analysis of a sequence of images acquired in a standard widefield or total internal reflection fluorescence (TIRF) microscope directly generates a super-resolution reconstruction without fluorophore detection and localization. However, contrary to other approaches, SRRF provides a single analytical framework that enables high-fidelity reconstructions for a wide variety of data sets, with the final resolution of the SRRF image dictated by the properties of the data set analysed. For example, super-resolution reconstructions at a resolution of at least 50 nm, comparable to current benchmark localization algorithms, are obtained for SMLM data. High-resolution reconstructions are also obtained for confocal microscopy data (80–98 nm) and widefield LED-illuminated data (103–106 nm). Moreover, super-resolution information at a temporal resolution of 1 s and a spatial resolution down to 60 nm can be extracted from live samples using conventional fluorophores, low-intensity illumination and as few as 100 raw frames.

## Results

### SRRF algorithm

**Figure 1:**
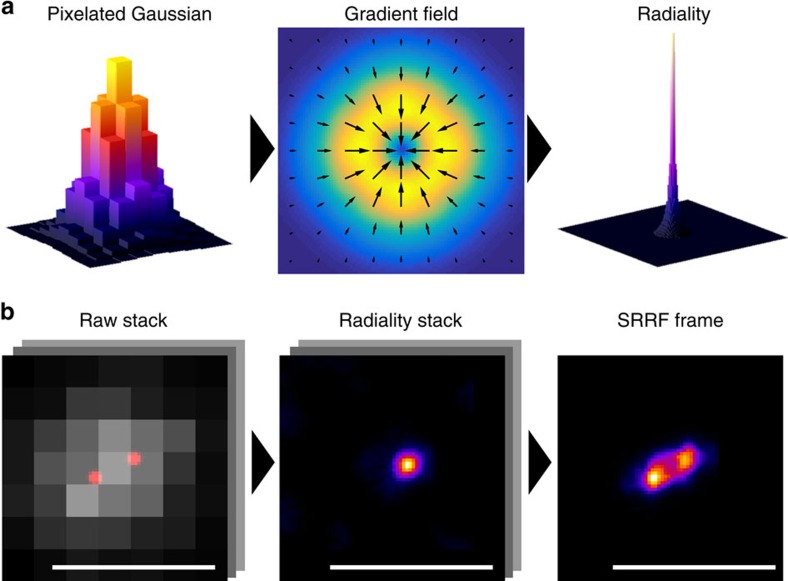
The SRRF algorithm. (**a**) Spatial analysis. Left: 3D surface plot of a pixelated simulated widefield PSF. Middle: surface plot of the gradient magnitude (arrows indicate direction). Right: 3D surface plot of the measured radiality PSF. (**b**) Temporal analysis. Left: a 100 frame simulation of a pair of fluctuating fluorophores separated by the sigma (σ) of the widefield PSF (135 nm). The true fluorophore positions are indicated in red and the pixelated simulated noisy intensity distribution is shown in grey. Middle: stack of radiality maps obtained by applying radiality to each image in the simulated image sequence. Right: SRRF image acquired by higher-order temporal analysis of the stack of radiality maps. Scale bar, 500 nm.

The SRRF algorithm is provided in the form of the NanoJ-SRRF software package (Supplementary Software), a freely available open-source plugin for the popular ImageJ or Fiji image analysis software^13^. SRRF is a fast, threshold free algorithm comprising temporal analysis of a sub-pixel geometrical measure applied to an image sequence (Fig. 1, Supplementary Note 1, Supplementary Figs 1 and 2). SRRF assumes the image is formed of point sources convolved with a point spread function (PSF) that displays a higher degree of local symmetry than the background. This assumption draws on similar geometric principles used by one of the most accurate low-density SMLM methods, radial symmetry^7^,^14^,^15^. Rather than detect and localize single radially symmetric points, however, for each frame in an image sequence SRRF calculates the degree of local gradient convergence (referred to here as radiality) across the entire frame, on a sub-pixel basis. Thus, information in the gradient field, which would otherwise be discarded by a localization technique, is maintained for further temporal analysis. In the case of a single fluorophore this results in a continuous conical distribution with a significantly reduced full-width-half-maximum (FWHM) that can be displayed on an up-sampled pixel grid (Fig. 1a). This radiality distribution is capable of distinguishing two Gaussian PSFs separated by ∼0.7 times the Gaussian FWHM (1.7 times the PSF s.d. σ; Supplementary Movie 1). In addition, the FWHM of this radiality distribution can be adjusted by changing the radius at which the gradient convergence is measured. The distribution is independent of PSF intensity and is robust to changes in PSF size. Furthermore, the radiality distribution preserves deformations of the PSF, for example astigmatic PSFs and polarization effects, without a displacement in the peak position. The radiality map of a full image will, however, include a number of non-fluorophore-associated radiality peaks, as transient local radial symmetries can occur in image noise. Weighting the radiality map by the fluorescence intensity of the acquired frame and local image gradient magnitudes allows the map to be de-noised without a significant increase in the FWHM of the radiality peaks. Further de-noising and contrast enhancement can be achieved by applying temporal analysis to generate a single super-resolution frame from the resulting radiality image sequence (Fig. 1b), which can also further enhance the resolution (Supplementary Note 1). This enhancement is based on two concepts. (a) Noise-induced radiality peaks are uncorrelated in time, thus a pixel-wise temporal correlation at their location will approach zero as the number of imaged frames increases. (b) The highest degree of temporal correlation is located at the centre of the radiality peaks generated by fluorophore signal. As in the case of SOFI^12^, higher-order temporal cumulants can be calculated on the radiality map further enhancing contrast and, in the case of low-density data sets, increasing resolution by reducing the FWHM of the radiality distribution. However, compared with SOFI, the undesirable non-linear response to brightness at higher-order cumulants^16^ is to a large extent alleviated in SRRF since the peak radiality of all fluorophores is similar and independent of the fluorophore brightness.

### *In silico* characterization of SRRF performance

**Figure 2:**
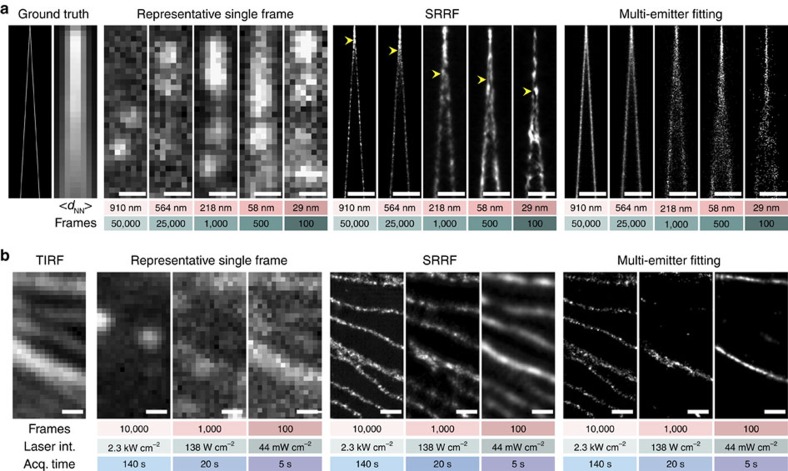
SRRF applied from low- to ultra-high-fluorophore densities on simulated and experimental data. (**a**) Simulations of multiple densities of emitting fluorophores, mean nearest neighbour distance <d_NN_>, and number of frames shown underneath equivalent images. Ground truth consists of two diverging lines separated by 0 nm (top) through to 350 nm (bottom) with fluorophores placed every 5 nm along the lines (further simulation parameters described in the ‘Methods' section). Left: ground truth, equivalent diffraction limited image and representative single-simulated frames. Middle: reconstructions from SRRF. Right: reconstructions from multi-emitter fitting with maximum-likelihood estimation. Yellow arrows on SRRF reconstructions indicate point at which filaments are no longer resolved (from left to right 30, 50, 100, 110 and 140 nm). Scale bars, 500 nm. (**b**) Fixed microtubules labelled with Alexa Fluor 647, imaged with different laser intensities to produce different length data sets of varying fluorophore densities. Number of frames in data set, on-sample laser intensity and total acquisition time shown underneath images. The same region of the sample was imaged under each set of conditions. Far left: TIRF image of region. Left: representative single frames from acquired data sets. Middle: reconstructions from SRRF. Right: reconstructions from multi-emitter fitting with maximum likelihood estimation. Scale bars, 500 nm.

We have validated the accuracy of the algorithm under a range of conditions using simulated data (Supplementary Note 2). At low densities, near-Cramér-Rao^8^ limited precisions are achieved (Supplementary Fig. 3) and SRRF achieves greater separation than multi-emitter fitting for constantly emitting fluorophore pairs exhibiting intensity fluctuations (Fig. 1b, Supplementary Fig. 4). In the case of many overlapping fluorophores the local radial symmetry is reduced and consequently the magnitude of the radiality is diminished; however, a conical distribution with a reduced FWHM is still observed parallel to any axis of gradient convergence allowing super-resolution of structures at ultra-high densities (defined here as a mean emitting fluorophore separation, *d*_NN_, <100 nm). This is demonstrated using simulated data with varying fluorophore kinetics resulting in different fluorophore densities (Fig. 2a). Using SRRF, at *d*_NN_ ∼910 nm it is possible to resolve two approaching filaments at resolutions comparable to localization (∼30 nm). It is also possible to achieve resolutions of the order of 130 nm at *d*_NN_ ∼29 nm, beyond the point where the assumptions on fluorophore density required for fitting are exceeded. As expected, fitting methods failed to reconstruct the structure at these ultra-high-fluorophore densities. Extensive quantitative evaluation of SRRF at ultra-high densities was performed by simulating parallel filaments separated by less than the resolution limit and calculating the normalized visibility^17^ of the filaments in the reconstructed images (Supplementary Note 2, Supplementary Fig. 5). The separation of the filaments, labelling density, fluorophore kinetics, brightness and background levels were varied. Supplementary Fig. 5 demonstrates that under the general conditions of long fluorophore on-times, leading to high densities of emitting fluorophores, SRRF outperforms both multi-emitter fitting and SOFI.

To confirm the results from analysis of simulated data, a low-density STORM data set was acquired using fixed Alexa Fluor 647 immuno-labelled microtubules and used to generate two high-density data sets through temporal averaging of 100 and 500 frame sequences. From these data sets we determined that two microtubules separated by ∼75 nm can be resolved at low densities (Supplementary Note 2, Supplementary Fig. 6). At ultra-high density (*d*_*NN*_=31 nm) both microtubules are still resolved albeit with lower visibility. Notably, the performance of SRRF on high-density data sets allows the production of a super-resolution image with as few as 100 raw frames, achievable in <1 s using current EMCCD cameras.

### Comparative performance at varying illumination intensities

Experimentally, the emitting fluorophore density can be modulated by varying the on-sample illumination intensity since lower intensities result in fewer transitions to transiently stable dark states^18^. Reconstructions of three examples of such data through SRRF and multi-emitter fitting are shown in Fig. 2b, again demonstrating the performance of SRRF across different densities. Here, by selecting three different laser illumination intensities, varying density data sets are produced in a manner analogous to the varying fluorophore kinetics simulations as shown in Fig. 2a. Dividing a raw image sequence for these data sets in half and applying SRRF provides two images on which Fourier ring correlation (FRC) can be performed. FRC measures the similarity of two images as a function of spatial frequency giving a quantitative estimate of the resolution^19^,^20^,^21^. As was the case for the SRRF reconstructions in Fig. 2a, resolution increased as density decreased; FRC analysis of the SRRF images in Fig. 2b indicated resolutions of 49 nm (low-density ‘LD' data, 2.3 kW cm^−2^), 105 nm (high-density ‘HD' data, 138 W cm^−2^) and 156 nm (ultra-high-density ‘UHD' data, 44 mW cm^−2^; Supplementary Fig. 7a). Using these three data sets we performed a further comparative analysis of SRRF with the two best performing, publicly available high-density localization algorithms^7^, DAOSTORM^9^ and FALCON^22^, and the image based methods deconSTORM^10^, 3B^11^ and SOFI^12^ (Supplementary Note 3). SRRF and deconSTORM were the only two algorithms able to produce fully representative reconstructions in all three data sets (Supplementary Fig. 8). Notably, SRRF analysis takes only a few minutes for most full-frame data sets due to the use of graphics processing unit-based high-performance computing approaches, at least 40-fold faster than the methods used for comparison here, with the exception of SOFI.

To assess the similarity of the reconstructed images line profiles were taken across the full extent of each reconstruction and a pairwise correlation between the profiles was performed (Supplementary Note 3, Supplementary Figs 9 and 10). The SRRF low-density reconstruction had the second highest correlation against the DAOSTORM low-density reconstruction and similarly high correlations were achieved for the high- and ultra-high-density SRRF reconstructions (Supplementary Fig. 10).

### Applicability to different imaging modalities

**Figure 3:**
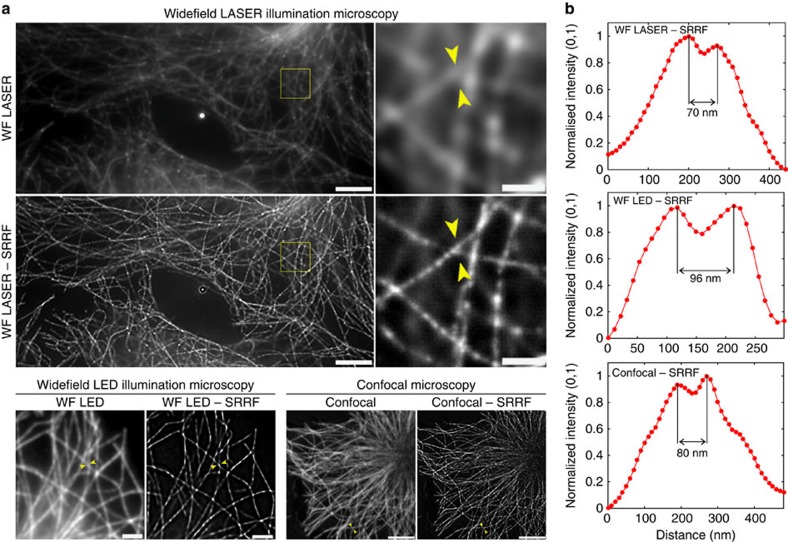
SRRF applied to widefield laser and LED illumination microscopy and confocal microscopy. (**a**) Widefield laser-illuminated image and SRRF reconstruction of a fixed cell with Alexa Fluor 647-labelled microtubules following dSTORM acquisition (scale bar 5 μm) and expanded view of the yellow-boxed region (scale bar, 1 μm); widefield LED-illuminated image and SRRF reconstruction of Alexa Fluor 647-labelled microtubules (scale bar, 2 μm); confocal image and SRRF reconstruction of Alexa Fluor 647-labelled microtubules (scale bar, 5 μm). (**b**) Normalized line profiles taken from the regions between the yellow arrowheads for corresponding SRRF images in **a** showing separated features.

To test the broad applicability of SRRF to different imaging modalities we acquired image sequences of Alexa Fluor 647 immuno-labelled microtubules under the following conditions: (i) high-intensity laser-illuminated widefield epifluorescence, generating a low-density STORM-like data set; (ii) low-intensity LED-illuminated widefield epifluorescence, generating an ultra-high-density data set; (iii) laser-scanning confocal, generating an ultra-high-density data set (Fig. 3). For all three imaging modalities samples were fixed using paraformaldehyde at 37 °C to preserve sample ultrastructure and thickness such that for the widefield images the effect of out-of-focus fluorescence on SRRF reconstructions could be ascertained. For example, in the SRRF reconstruction of the laser widefield data set (Fig. 3a) resolution and contrast are significantly enhanced for in-focus structures, whereas out-of-focus structures are dimmer and more blurred. Analysis of peak-to-peak separations of microtubules identified in these images indicates achievable resolutions of the order of 70, 96 and 80 nm for each experimental condition, respectively (Fig. 3b). The resolutions obtained in the confocal and LED widefield images were further confirmed using FRC analysis (Supplementary Fig. 7b,c).

### Live-cell super-resolution using SRRF

The performance of SRRF at high-fluorophore densities enables the use of fast, low-illumination intensity imaging regimes compatible with conventional fluorophores in living cells. We demonstrate this versatility by super-resolving the dynamics of stably transfected tubulin-GFP HeLa cells at 1 super-resolution frame per second (f.p.s.) at resolutions of the order of 60 nm, as measured by peak-to-peak separations, to 120 nm, as measured by FRC analysis (Supplementary Movie 2, Supplementary Figs 7d and 11a). The apparent discrepancy between the FRC and peak-to-peak resolutions is likely to arise from the motion of the live-cell sample in this data set. Due to this motion the two images used for FRC are not of identical structures and as such a global measure of resolution will be reduced in comparison with a local measure applied to a single image.

The use of low laser powers (2 × 10^−4^ kW cm^−2^ to 2 × 10^−1^ kW cm^−2^), well below the 1–20 kW cm^−2^ range traditionally used in PALM or STORM approaches^23^ (Supplementary Table 1), allows long-term imaging without visual signs of phototoxic effects as highlighted in a recent study^24^ quantifying the relationship between irradiation intensity and cell-survival probability for super-resolution imaging. This has enabled time-lapse super-resolution imaging with 1 s frames at 25 min intervals for up to 24 h. Supplementary Movie 3 shows a SRRF reconstruction of one such acquisition over an 8-h period in which a tubulin-GFP expressing HeLa cell undergoes division, demonstrating that phototoxicity is sufficiently low for mitosis to proceed while achieving image resolutions of the order of 106 nm (peak-to-peak separation, Supplementary Fig. 11b). To demonstrate that this capability is independent of fluorophore type and not dependent on 1-dimensional filamentous structures, we performed super-resolution imaging at 1 f.p.s. of mitochondria using the fluorescent dye, MitoTracker Red, demonstrating resolutions of the order of 165 nm via FRC analysis (Supplementary Movie 4, Supplementary Fig. 7e).

### Imaging actin cortex dynamics in the immunological synapse

**Figure 4:**
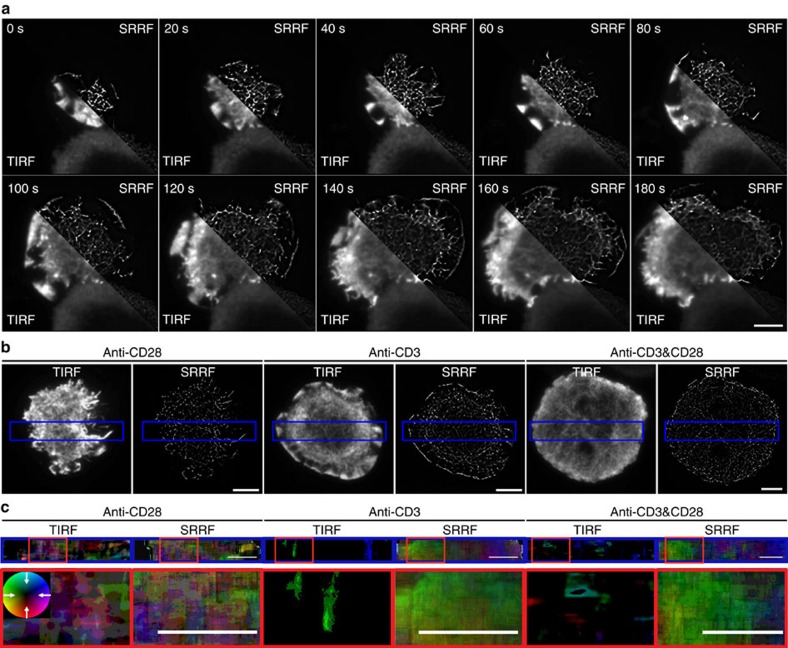
SRRF used for super-resolution live-imaging of Jurkat T cells transiently expressing LifeAct-GFP. (**a**) Conventional TIRF microscopy (TIRF) versus SRRF images (SRRF) of Jurkat T cells transfected with LifeAct-GFP dropped on an anti-CD3-coated coverslip and imaged for 180 s at 1 super-resolution f.p.s. (**b**) Conventional TIRF and SRRF images of Jurkat T cells expressing LifeAct-GFP imaged on coverslips coated with anti-CD28 alone, anti-CD3 alone or in combination (anti-CD3 and -CD28) to stimulate an immunological synapse formation (highlighted area corresponds to the region used for PIV analysis). (**c**) PIV analysis of corresponding Supplementary Movies 6–8 shows notable retrograde actin flow in anti-CD3 but not in anti-CD28 stimulated Jurkat T cells. A colour-coded measure of flow directionality and speed is plotted for the blue highlighted regions. White arrows in colour wheel are representative of flow direction, central colour (minimum intensity) corresponds to 0 μm min^−1^, saturated colours (maximum intensity) correspond to 38.4 μm min^−1^. Scale bars, 5 μm.

The ability of SRRF to produce super-resolution images with temporal resolution of 1 s allows for detailed imaging of highly dynamic biological processes. One example is the rapid remodelling of the actin cortex during the formation of an immunological synapse^25^. We imaged Jurkat T cells transfected with LifeAct-GFP dropped onto a coverslip coated with antibodies to emulate the formation of a synapse after T-cell receptor engagement (Supplementary Movie 5; Fig. 4a). This system produces a significant challenge for image reconstruction with conventional algorithms since the density of the actin varies significantly across the synapse. SRRF allows for resolution of the sub diffraction scale structure of the cortex, not otherwise discernible in conventional TIRF images.

We used particle image velocimetry (PIV)^13^ to demonstrate the degree of improvement afforded by using SRRF, in contrast to the corresponding TIRF images, in characterizing the movement of the actin cortex. We have previously measured the retrograde flow of the actin cortex in the periphery of T-cell synapses using molecular flow algorithms applied to SIM images^25^. Here, using PIV applied to SRRF reconstructions we compared the dynamics of the actin cortex of Jurkat T cells expressing LifeAct-GFP imaged on anti-CD28, anti-CD3 and both anti-CD3- and CD28-coated coverslips (Supplementary Movie 6–8; Fig. 4b). Single-frame reconstructions using deconvolution, SOFI, deconSTORM and 3B are provided for comparison in Supplementary Fig. 12 (Supplementary Methods). PIV revealed the velocity field generated by the actin dynamics indicating the directionality and relative speed (encoded in different colours) of actin in the different stimulatory conditions. We show that stimulation by anti-CD28 alone does not induce retrograde flow of the actin cortex with both the TIRF and SRRF movies exhibiting a random velocity field (Fig. 4c). In contrast, analysis of SRRF movies shows that both anti-CD3 stimulation and anti-CD3 and CD28 co-stimulation induce retrograde flow of the actin cortex in the periphery of the synapse, an effect not accurately quantifiable when PIV analysis is applied to the TIRF data (Fig. 4c). Resolutions down to 64 nm were measured through peak-to-peak separation of actin structures in the anti-CD3 and CD28 movie (Supplementary Fig. 11c, Supplementary Movie 8).

## Discussion

We have shown here that SRRF is a unique and highly versatile technique for both fixed-cell and dynamic live-cell super-resolution using conventional fluorophores, which is also compatible with a variety of imaging modalities. This is in contrast to methods such as SIM, which although compatible with live-cell imaging requires expensive optics, and SMLM, which is broadly limited to imaging with phototoxic laser intensities. We have also demonstrated that, compared with other super-resolution algorithms capable of analysing high-density data sets, SRRF provides a significant reduction in reconstruction artefacts, such as the disappearance of structures or ringing effects while remaining computationally efficient. As such the flexibility of SRRF analysis across a range of microscopes and sample preparation methods, along with its distribution as a freely available Fiji plugin (a model previously used for the popular SMLM software QuickPALM^26^), allows virtually any laboratory access to super-resolution imaging.

## Methods

### Cell lines

HeLa and LLC-MK2 cells were kindly provided by Dr Mark Marsh, MRC-LMCB, UCL. HeLa H2B-mCherry/mEGFP-α-tubulin stable cell line^27^ was kindly provided by Dr Buzz Baum, MRC-LMCB, UCL. CHO cells were kindly provided by Dr Jason Mercer, MRC-LMCB, UCL. Dr Dylan Owen's, KCL, Jurkat T cells (E6.1) were obtained from APCC. Cell lines were not authenticated. All cell lines (except Jurkats that were not tested) tested negative for mycoplasma (MycoAlert, Lonza).

### Sample preparation for TIRF imaging of fixed microtubules

LLC-MK2 cells were cultured in phenol red-free Dulbecco's modified Eagle's medium (DMEM; Gibco) supplemented with 10% fetal bovine serum (FBS; Gibco) and 1% penicillin/streptomycin (Sigma) at 37 °C in a 5% CO_2_ incubator. Cells were seeded on ultra-clean^28^ 13 mm diameter thickness #1.5 coverslips at a density of 0.1 × 10^6^ per 35 mm dish. Fixation was performed using ice-cold methanol for 10 min followed by washing with cytoskeleton stabilizing buffer (CSB; 60 mM PIPES, 25 mM HEPES, 10 mM EGTA and 2 mM MgCl_2_ at pH 6.9 in phosphate-buffered saline (PBS)). Additional permeabilization was performed (0.05% Triton X-100 in CSB) for 5 min followed by three washing steps using 0.05% Tween-20 in CSB and blocking in 5% bovine serum albumin (BSA; Sigma) for 40 min.

Microtubules were stained using mouse monoclonal anti-α-tubulin antibodies (clone DM1A, Sigma) at a dilution of 1:500 in 5% BSA for 1 h and then goat anti-mouse secondary antibodies conjugated to Alexa Fluor 647 (Life Technologies) at a dilution of 1:1,000 in 5% BSA for 45 min. A secondary fixation step was performed with 2% PFA in CSB for 10 min. For drift correction, 100 nm TetraSpeck beads (Life Technologies) were added at a dilution of 1:1,000 in PBS for 10 min to each coverslip. Coverslips were mounted on clean microscope slides^28^ in 100 mM mercaptoethylamine (MEA; Sigma) at pH 7.4 and all imaging was performed within 3 h of mounting.

### Sample preparation for widefield and confocal imaging

For epifluorescence (with laser and LED illumination) and confocal microscopy imaging, CHO cells were cultured in phenol red-free Minimum Essential Medium Alpha (MEMα; Gibco) supplemented with 10% FBS (Gibco) and 1% penicillin/streptomycin (Sigma) at 37 °C in a 5% CO_2_ incubator. Cells were seeded on ultra-clean^28^ 8 mm diameter thickness #1.5 coverslips (Zeiss) at a density of 0.1 × 10^6^ per 35 mm dish. To preserve cell ultrastructure fixation was performed with 4 % paraformaldehyde (PFA) in a modified version of CSB (5 mM KCl, 0.1 mM NaCl, 4 mM NaHCO_3_, 11 mM Na_2_HPO_4_, 2 mM MgCl_2_, 5 mM PIPES, 2 mM EGTA, pH 6.9) for 15 min at 37 °C, followed by washing with the same buffer (without PFA). Additional permeabilization was performed (0.05% Triton X-100 in CSB) for 5 min followed by three washing steps using 0.05% Tween-20 in the modified version of CSB and blocking in 5% BSA (Sigma) for 40 min. Microtubules were stained using mouse monoclonal anti-α-tubulin antibodies (clone DM1A—T6199, Sigma) at a dilution of 1:1,000 (widefield LED illumination and confocal microscopy) or 1:2,500 (epifluorescence) in 5% BSA for 1 h and then goat anti-mouse secondary antibodies conjugated to Alexa Fluor 647 (Life Technologies) at a dilution of 1:1,000 in 5% BSA for 45 min. A secondary fixation step was performed with 2% PFA in PBS for 10 min. For drift correction, 100 nm TetraSpeck beads (Life Technologies) were added at a dilution of 1:1,000 in PBS for 10 min to each coverslip. Coverslips were mounted on clean microscope slides^28^ in 100 mM mercaptoethylamine (Sigma) at pH 7.3 and all imaging was performed within 3 h of mounting.

### Sample preparation for live-cell imaging of HeLa cells

HeLa cells and HeLa H2B-mCherry/mEGFP-α-tubulin stable cell line^27^ were grown in DMEM containing 10% FBS, 100 U ml^−1^ penicillin and 100 μg ml^−1^ streptomycin at 37 °C with 5% CO_2_ in a humidified incubator. Before imaging cells were seeded on to #1.5 glass bottom 35 mm μ-Dish (ibidi GmbH); for mitochondrial imaging HeLa cells were seeded in 8-well glass bottom μ-Slides (ibidi GmbH) and stained with MitoTracker Red CM-H_2_Xros (Molecular Probes) following the manufacturer's recommendations.

### Sample preparation for live-cell imaging of Jurkat T cells

Jurkat T cells were cultured in Roswell Park Memorial Institute (RPMI) 1640 media (Gibco) supplemented with 10% fetal calf serum (Life Technologies) and 1% penicillin/streptomycin (Sigma) at <5 × 10^5^ cells ml^−1^ at 37 °C, in a 5% CO_2_ atmosphere. For transfection, T cells were split 24 h before electroporation to ensure cells were in log growth phase. On the day of transfection 2 × 10^7^ cells were pelleted at 238 *g*, resuspended in OptiMem (Life Technologies) pre-equilibrated to 37 °C, pelleted a second time at 238 *g* and resuspended in 500 μl fresh OptiMem in a cuvette (BioRad) with 5 μg of LifeAct-GFP plasmid (ibidi GmbH). Electroporation for Jurkat cells was carried out on a Gene Pulser Xcell system (BioRad) set to an exponential decay pulse using 300 V and 950 Ω capacitance. After pulsing, viable cells were separated from dead matter and reseeded in 5 ml pre-equilibrated RPMI1640 media overnight.

To stimulate Jurkat T cells into producing an immunological synapse, #1.5 8-well chamber coverslips (Labtek) were coated with 1 μg ml^−1^ of either anti-CD3 alone, anti-CD28 alone or anti-CD3 and CD28 (Cambridge Biosciences and BD Bioscience) in PBS, added to the glass coverslip surface overnight at 4 °C. In all, 18–24 h after electroporation the antibody-coated coverslips were washed 1 × in 200 μl pre-equilibrated 1 × Hank's balanced salt solution (HBSS) with 20 mM HEPES before imaging and placed on the microscope. In all, 100 μl of cell-containing media was pelleted and resuspended in 200 μl 1 × HBSS (+20 mM HEPES). In all, 100 μl of Jurkat T cells were then pipetted into one of the stimulatory coverslip wells resulting in a final concentration of 5 × 10^5^ cells ml^−1^.

### Imaging

TIRF imaging of fixed microtubules was performed using a Nikon N-STORM inverted optical microscope in TIRF mode. A 100 × TIRF objective (Plan-APOCHROMAT 100 × /1.49 Oil, Nikon) was used with additional 1.5 × magnification to collect fluorescence onto an EMCCD camera (iXon Ultra 897, Andor), yielding a pixel size of 107 nm, and excitation was provided by a 647 nm laser. Imaging conditions for the data sets used in Fig. 2b and Supplementary Fig. 8 were as follows. Ultra-high density: 100 frames acquired with 1% laser power (equivalent to ∼44 mW cm^−2^ at the sample) and 50 ms exposure time. High density: 1,000 frames acquired with 15% laser power (equivalent to ∼138 W cm^−2^ at the sample) and 20 ms exposure time. Low density: 10,000 frames acquired with 100% laser power (equivalent to ∼2.3 kW cm^−2^ at the sample) and 14 ms exposure time.

Epifluorescence imaging of fixed microtubules was performed using an ElyraPS.1 inverted microscope. For excitation a 642 nm laser operating at 100% power (equivalent to ∼4 kW cm^−2^ at the sample) was used to induce blinking behaviour in Alexa Fluor 647. A 100 × TIRF objective (Plan-APOCHROMAT 100 × /1.46 Oil, Zeiss) was used, with additional 1.6 × magnification, to collect fluorescence onto an EMCCD camera (iXon Ultra 897, Andor), yielding a pixel size of 100 nm. Cropped areas were imaged to allow for fast imaging at 50 f.p.s.

LED illumination imaging of fixed microtubules was performed using a Nikon N-STORM inverted optical microscope in EPI mode. Excitation was provided by a 647 nm LED illumination source operating at 100% power. A 100 × TIRF objective (Plan-APOCHROMAT 100 × /1.49 Oil, Nikon) was used with additional 1.5 × magnification to collect fluorescence onto an EMCCD camera (iXon Ultra 897, Andor), yielding a pixel size of 107 nm. Cropped areas were imaged to allow for fast imaging at 96 f.p.s.

Confocal microscopy imaging of fixed microtubules was performed using a Leica TCS SP8 STED 3 × confocal microscope equipped with a STED immersion oil objective (HC PL APO 100 × /1.40 Oil, Leica). Excitation was provided by a 647 nm white light laser (WLL, 470–670 nm) at 100% laser power. Cropped areas and magnification were chosen to allow for fast imaging at 50 f.p.s. and a final pixel size of 100 nm.

Long-term live-cell imaging of mEGFP-Microtubules in HeLa H2B-mCherry/mEGFP-α-tubulin stable cell line was performed in DMEM containing 10% FBS at 37 °C with 5% CO_2_ in a humidified incubator using the N-STORM inverted microscope (Nikon) in TIRF mode as described above. The microscope was programmed to automatically acquire 100 frames every 25 min for 24 h, with ∼0.65 kW cm^−2^ on-sample 488 nm laser intensity and a pixel size of 107 nm.

Short-term imaging of microtubule dynamics was performed using the same sample preparation and using an ElyraPS.1 inverted microscope (Zeiss) in TIRF mode. This provided ∼0.009 kW cm^−2^ on-sample 488 nm laser intensity for continuous imaging with a pixel size of 100 nm.

Live-cell imaging of mitochondria was performed in DMEM containing 10% FBS at 37 °C with 5% CO_2_ in a humidified incubator using the ElyraPS.1 inverted microscope in HiLO mode. A 561 nm excitation laser was angled through the back focal plane of a 100 × TIRF objective (Plan-APOCHROMAT 100 × /1.46 Oil, Zeiss), providing ∼0.0003, kW cm^−2^ on-sample intensity. Emitted signal from the excited dye molecules was collected by an EMCCD camera (iXon 897, Andor), with a pixel size of 100 nm.

Live-cell imaging of Jurkat T cells expressing LifeAct-GFP during synapse formation was performed using an N-STORM inverted microscope (Nikon) in TIRF mode. A 488 nm excitation laser was angled through the back focal plane of a 100 × TIRF objective (Nikon). A range of laser powers were tested for imaging the samples; as GFP intensity fluctuations are minimal regardless of laser power in the absence of a specialized imaging buffer, similar SRRF reconstructions were obtained at all powers tested. As a result, all T-cell images displayed were obtained with the lowest laser power tested. A total of 100% laser power at the fibre end constitutes 80 mW (∼0.83 kW cm^−2^ at the sample), and intensities used for individual acquisitions are shown in Supplementary Table 1. Emitted signal from the excited GFP molecules was collected by an EMCCD camera (iXon Ultra 897, Andor), with a pixel size of 160 nm.

### PIV analysis

SRRF and interpolated raw data image stacks (to match pixel size and temporal resolution) were analysed using the PIV analyser Versus1.2 using a 64 × 64 pixel window and with interpolation to obtain sub-pixel flow magnitudes. The resulting colour-coded velocity field stack was averaged to obtain the final PIV result.

### Image processing for movies

For all live-cell movies, images were acquired at 100 f.p.s. SRRF movie frames were produced by running SRRF analysis on groups of 100 frames to yield super-resolution frame rates of 1 f.p.s. To generate comparable TIRF movies, each TIRF movie frame was created by averaging the same 100 frames as used to render each SRRF movie frame. Intensity normalization was applied to the TIRF and SRRF movie frames as the last stage following all other analyses for display.

### *In silico* generation of high-density data sets

Imaging was performed as described for fixed microtubules using an N-STORM microscope in TIRF mode with 100% 647 nm laser illumination (2.3 kW cm^−2^ on-sample intensity) to obtain sparse, low-density data sets. Data sets consisting of 100,000 frames were acquired and subsequently truncated to only the later frames where the number of emitters per frame was constant (first 1,000 frames rejected for data shown in Supplementary Fig. 6). Each frame in the high-density data set was produced by averaging 100 frames of the low-density data set (that is, frame 1 of the high-density data set is the average of frames 1–100 of the low-density data set, frame 2 is the average of frames 101–200 of the low-density data set and so on), and each frame in the ultra-high-density data set was produced by averaging 500 frames of the low-density data set. This will reduce the variance of the background by a factor of √n, where n is the number of frames averaged, and hence increases the signal-to-noise ratio. To avoid alterations in noise statistics impacting on the multi-emitter fitting performed, weighted least squares fitting was used as opposed to maximum likelihood estimation as this is less sensitive to the noise model. ThunderSTORM was used to localize particles in the low-density data set and these localizations were then used to estimate the average distance of each fluorophore to its nearest neighbour within the same frame (*d*_NN_ values^7^) for all three data sets presented in Supplementary Fig. 6. This approach provided the advantages of both knowing the fluorophore density and also providing a ‘ground truth' for experimental data as obtained from the ThunderSTORM reconstruction of the low-density data set against which high-density reconstructions could be compared.

### Simulated data

Simulated images used in the evaluation of SRRF were produced by modelling the PSF of a single emitter as a Gaussian distribution with a standard deviation of 135 nm. Rendering of the simulated images was performed on a 10 nm high-resolution lattice and integrated to form a 100 nm pixel grid representing the CCD camera pixels. The emission rate of individual fluorophores was randomly distributed with a standard deviation of 40% of the mean emission rate in each frame. A background of 100 photons was added to all pixels before adding Poisson distributed photon shot noise. The EMCCD conversion of photons to pixel values was simulated using 2 photoelectrons per A/D count and a base level of 100 A/D counts with a frame rate 30 f.p.s. A linear EMCCD gain of 100 was applied and a read noise of mean 10, standard deviation 3 added. The ground truth structure in Fig. 2a consists of two lines separated by 0 nm at the top and 350 nm at the bottom of the frame with emitters evenly distributed at a separation of 5 nm along the lines. The positions of emitters in the single-frame simulations used in Supplementary Fig. 3 were randomly distributed within a 100 nm pixel. The central positions of emitter pairs in the simulations used in Supplementary Fig. 4 were randomly distributed within a 100 nm pixel.

Temporal characteristics of emitters in Fig. 2a were modelled using a time continuous two state (emitting, non-emitting) Markov process not synchronized with the frame rate giving exponentially distributed on-times and off-times. The characteristic on-time was varied from 0.6 to 0.1 s, while the characteristic off-time was varied from 6.6 to 80.0 s giving on-state probabilities of 1/12, 3/80, 3/400, 1/400 and 1/800 leading to the various mean emitter separations quoted in Fig. 2a. The mean photon emission rate of the emitters was varied empirically to keep the single-frame peak signal-to-noise ratio approximately constant at 10 independent of density.

Simulations for the visibility analysis consist of two 2 μm long filaments, each 0.02 times the FWHM wide. The FWHM of the PSF was set to 317 nm. The separation of the filaments was varied from 0.1 to 1.1 times the FWHM (31.7 to 348.7 nm). The fluorophores were placed randomly on the filaments with a mean labelling density, which was varied from 1 to 21 μm^−1^. The ratio of the off rate, *k*_off_, of the fluorophores to the on rate, *k*_on_, *r*=*k*_off_/*k*_on_ was varied from 0.1 to 10. The mean fluorophore emission rate was varied from 100 photons s^−1^ to 10,000 photons s^−1^, while the background was varied from 0 to 50% of the mean photon emission rate. The mean fluorophore switching rate *k*=*k*_on_*k*_off_/(*k*_on_+*k*_off_) was kept constant at half the frame rate of 1 f.p.s. Rendering was performed as described above.

### Visibility analysis

To quantify the quality of the super-resolution reconstructions of parallel filaments a normalized visibility similar to that described in Geissbuehler *et al*.^17^ was calculated as follows. An average projection was taken of the reconstructed images parallel to the simulated filaments (Supplementary Fig. 5a) or a line profile perpendicular to reconstructed images microtubules as indicated in Supplementary Fig. 6a. Three intensity measures were taken from the resulting profile: the central value corresponding to the midpoint between the filaments, *I*_min_, and the intensities at the known filament positions, *I*_max,1_, *I*_max,2_. The normalized visibility was defined as


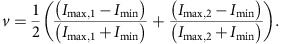


Super-resolution reconstructions of the simulations were performed using thunderSTORM, SOFI and SRRF. The best visibility measured using SOFI orders 1–6 and 4th order bSOFI was recorded.

### Determination of image resolution

To quantify image resolution through peak-to-peak separations of filamentous structures (Fig. 3 and Supplementary Fig. 11), line profiles were plotted from SRRF reconstructions between the yellow arrowheads as displayed. Each line profile was produced by averaging across an area ∼100 nm wide adjacent to the line to ensure that plots were not contaminated by random noise peaks. Line profiles were selected from the images such that resolution could be measured according to the Sparrow criterion^29^ whereby two structures are considered resolved once a noticeable dip is observed between their intensity profiles.

Quantification of the resolution by FRC was performed using a modified version of the Fourier Image REsolution algorithm^20^. For fixed samples (Figs 2b and 3) raw image sequences were divided in half (in time) and SRRF reconstructions were performed on each half separately. The largest possible power of 2 square regions of each data set (not containing fiducial markers which can bias FRC results) was selected to perform FRC. Each SRRF reconstruction was Fourier-transformed and concentric rings with a width of one pixel in Fourier space were segmented. The normalized cross correlation between corresponding rings in the two Fourier-transformed images was plotted against the spatial frequency corresponding to the radius of the rings. The resolution was determined as the point at which the FRC first falls below a threshold of 1/7 as previously used in Fourier Image REsolution^20^. A smoothed curve using local regression (LOESS) with bandwidth 0.0707 and robustness 0 was calculated for display only, threshold crossing was determined using the non-smoothed FRC. For live samples (Supplementary Movie 1 and 3) FRC was calculated using the first and second SRRF reconstructions in the movie and otherwise analysed as described above. The substantial movement of the sample between frames in Supplementary Movies 3 and 5–8 precluded the use of FRC on these data sets.

### Code availability

The source code used for SRRF analysis included in this study is provided within the article (Supplementary Software) and can be found at https://bitbucket.org/rhenriqueslab/nanoj-srrf. Source code for simulations and FRC analysis is available from the corresponding author on request.

### Data availability

Source data for Fig. 2b and Supplementary Figs 8 and 9 are provided at https://bitbucket.org/rhenriqueslab/nanoj-srrf/wiki/NanoJ%20sample%20data. All other data that support the findings of this study are available from the corresponding author on request.

## Additional information

**How to cite this article:** Gustafsson, N. *et al*. Fast live-cell conventional fluorophore nanoscopy with ImageJ through super-resolution radial fluctuations. *Nat. Commun.* 7:12471 doi: 10.1038/ncomms12471 (2016).

## Supplementary Material

Supplementary Information Supplementary Figures 1-12, Supplementary Tables 1- 2, Supplementary Notes 1-3, Supplementary Methods, Supplementary References.

Supplementary Movie 1 Rendering of the radiality transform for various parameters and input point-spread-functions. The radiality transform is shown for the real image (i.e. diffraction-limited image) under a wide range of conditions. In the first half of the movie the radiality transform of an image of a single fluorophore is considered. Firstly, the ring radius over which the radiality transform is calculated is varied from r = 0.1s - 3.00s (where s is the standard deviation of the PSF) and the corresponding radiality transform (right) is depicted for the diffraction-limited intensity profile shown on the left (frames 1-60). Next (frames 61-104), the ellipticity of the real intensity profile is varied by varying the sigma in x from sx = 0.5 - 2.0. The width of the PSF of the real intensity profile is then varied symmetrically from s = 1.0 - 2.0 (frames 105- 136). Finally, the intensity of the real image is varied from I = 0.5 - 30.0 a.u. (frames 137- 146). The second half of the movie shows the response of the radiality transform to images of two closely-positioned fluorophores. Firstly, the distance between the two fluorophores is increased from 0s - 8s (by changing the locations of the two fluorophore centers, xc1 and xc2, frames 147-230). The separation between the two fluorophores is then fixed at 1.8s and the intensity of one fluorophore is varied from I1 = 0 - 1 a.u. while the intensity of the other fluorophore I2 remains fixed at 0.5 a.u. (frames 231-274). In frames 275-323 the two fluorophores are rotated around each other to demonstrate invariance of the radiality transform to rotation, and also to provide a view from all angles of the transform. Finally, the fluorophores are moved back together so that they are perfectly overlapping (frames 324- 342).

Supplementary Movie 2 Live-cell microtubule dynamics imaged with SRRF. EGFP-labeled microtubules in live HeLa cells imaged at 100 frames per second, yielding 1 super resolution frame per second. First half: top panel TIRF imaging, bottom panel SRRF rendering, inset corresponds to zoom for second half of the movie. Second half: expansions of inset, plot shows the normalized intensity profiles along the yellow lines marked in the expanded TIRF and SRRF movies. Scale bar is 5 μm throughout.

Supplementary Movie 3 Long-term SRRF imaging of microtubule dynamics. EGFP-labeled microtubules in live HeLa cells imaged at 100 frames per second, yielding 1 super resolution frame per second, every 25 minutes for 8 hours. Top panel: TIRF imaging, bottom panel: SRRF rendering. Between 5 and 6 hours the cell lifts from the coverslip, undergoing mitosis. Scale bar is 5 μm.

Supplementary Movie 4 Mitochondrial dynamics imaged with SRRF. Mitotracker Red-labeled mitochondria in live HeLa cells imaged at 100 frames per second, yielding 1 super resolution frame per second. Top panel: TIRF movie, bottom panel: SRRF rendering. Scale bar is 5 μm.

Supplementary Movie 5 T cell spreading following drop imaged with SRRF. LifeAct-GFP-labeled actin in a live Jurkat T cell imaged at 100 frames per second, yielding 1 super resolution frame per second. The T cell is dropped onto an anti-CD3 coated coverslip, leading to spreading of the cell and reorganization of the intracellular actin network. Left panel: TIRF movie, right panel: SRRF rendering. Scale bar is 5 μm.

Supplementary Movie 6 Non-directed actin rearrangement following stimulation with anti-CD28 imaged with SRRF. LifeAct-GFP-labeled actin in a live Jurkat T cell imaged at 100 frames per second, yielding 1 super resolution frame per second. An anti-CD28 coated coverslip is used to stimulate immunological synapse formation. Left panel: TIRF movie, right panel: SRRF rendering. Scale bar is 5 μm.

Supplementary Movie 7 Retrograde flow of actin during immunological synapse formation upon stimulation with anti-CD3 imaged with SRRF. LifeAct-GFP-labeled actin in a live Jurkat T cell imaged at 100 frames per second, yielding 1 super resolution frame per second. An anti-CD3 coated coverslip is used to stimulate immunological synapse formation. Left panel: TIRF movie, right panel: SRRF rendering. Scale bar is 5 μm

Supplementary Movie 8 Retrograde flow of actin during immunological synapse formation upon stimulation with anti-CD3&CD28 imaged with SRRF. LifeAct-GFP-labeled actin in a live Jurkat T cell imaged at 100 frames per second, yielding 1 super resolution frame per second. An anti-CD3&CD28 coated coverslip is used to stimulate immunological synapse formation. Left panel: TIRF movie, right panel: SRRF rendering. Scale bar is 5 μm.

Supplementary Software The SRRF algorithm. The SRRF algorithm source code and plugin for ImageJ and Fiji are provided along with a manual providing instructions for installation and use.
