# Supplementary material for: Fast live-cell conventional fluorophore nanoscopy with ImageJ through super-resolution radial fluctuations
Source: Nat Commun. 2016 Aug 12;7:12471. doi: 10.1038/ncomms12471 (PMC4990649; doi:10.1038/ncomms12471)
Supplement: Supplementary Information — Supplementary Figures 1-12, Supplementary Tables 1- 2, Supplementary Notes 1-3, Supplementary Methods, Supplementary References. [file ncomms12471-s1.pdf]

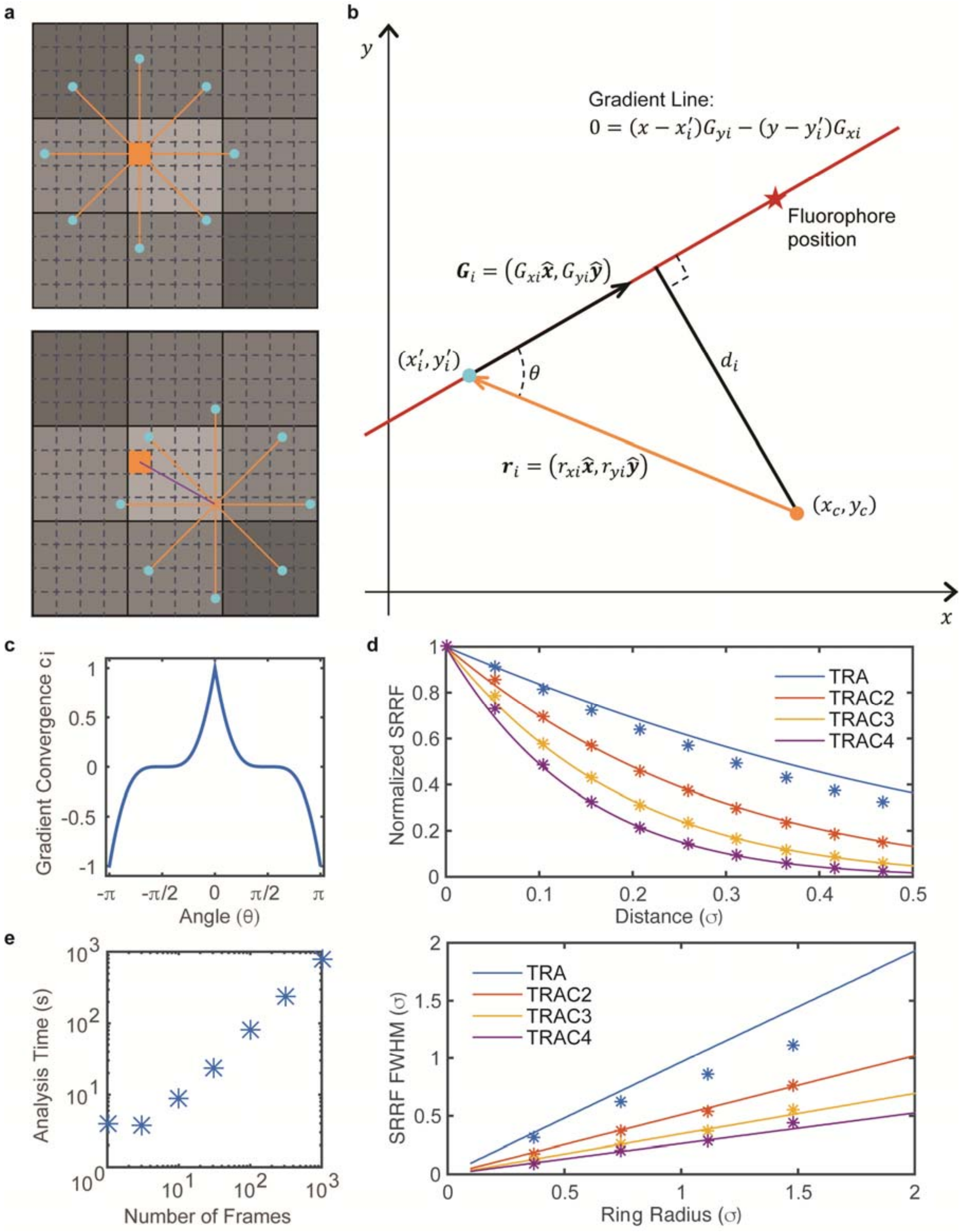

Supplementary Figure 1. The SRRF algorithm. (a) Top: Representation of the sub-pixel grid (dashed lines) with magnification 4 overlaid on the original image pixel grid. Location of radiality calculation sub-pixel (x, y), the N ring-coordinates and the ring radius are indicated (orange pixel, blue circles and orange lines). Bottom: The calculation centre (x_c, y_c) for the orange pixel is translated by the drift vector (purple line). **(b)** Illustration of the geometry for the calculation of d_i for a single ring coordinate (x'_i, y'_i). **(c)** Dependence of the gradient convergence, c_i, on θ. **(d)** (i) SRRF line profiles from the centre of a fluorophore; (ii) SRRF FWHM at varying ring radius; analysed with different temporal methods. Solid lines represent the theoretical distributions. Stars represent (i) the radially averaged profiles, (ii) the FWHM, of N=100, 100 frame simulated fluorophore fluctuation data sets SNR = infinity. **(e)** Dependence of analysis run time on number of 512x512 pixel frames for a 2013 2.4GHz Intel i7-4700MQ notebook processor with integrated graphics card.

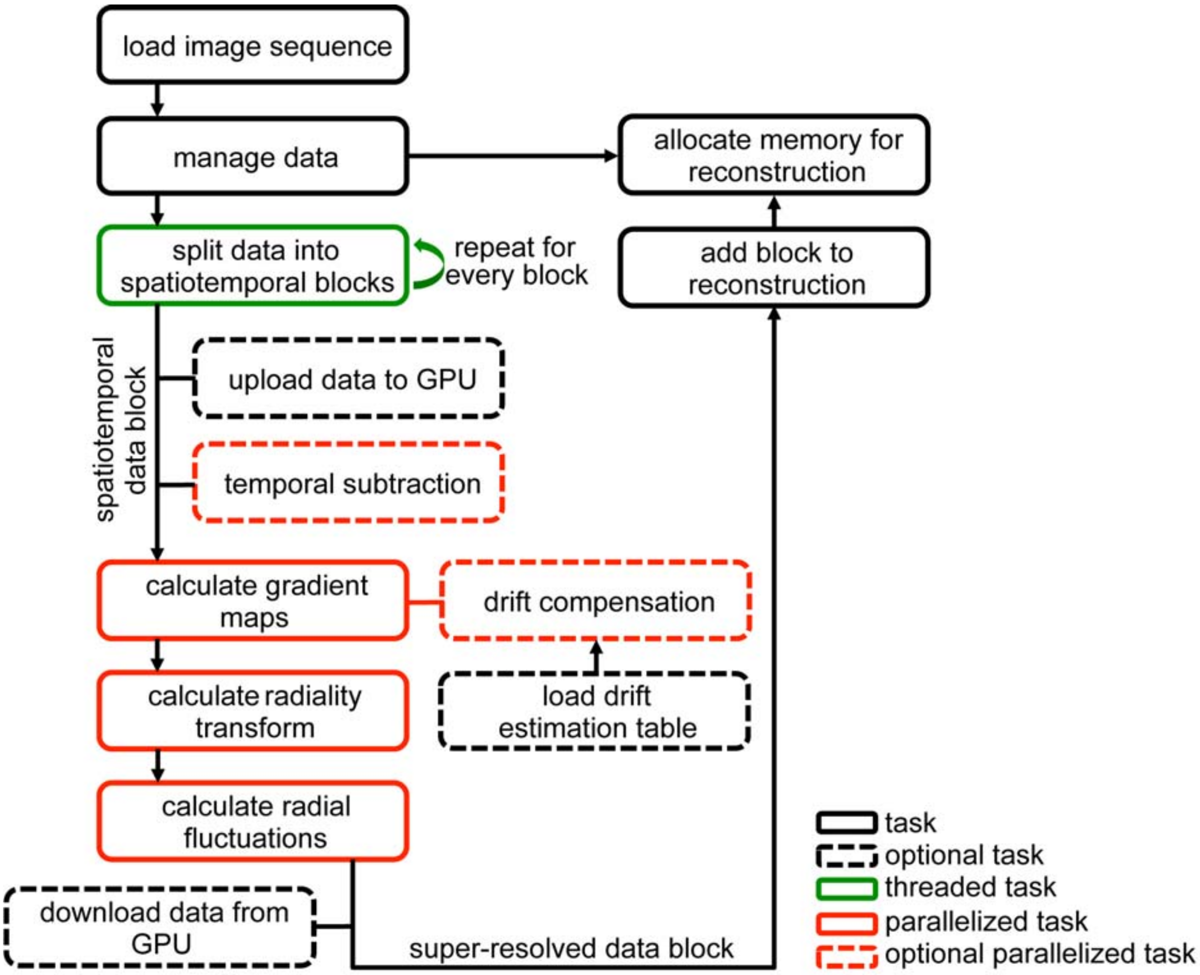

Supplementary Figure 2. Flow chart of the SRRF algorithm. Depiction of the main tasks carried by SRRF. Arrows indicate direction of task sequence, bottom right legend indicates task type. Of note, tasks labeled as parallelized are executed in OpenCL, while tasks labeled as threaded are executed in Java threads. Task "calculate gradient maps" is based on equation S2, task "calculate radiality transform" is based on equations S3-9 and task "calculate radial fluctuations" is based on equations S11-12.

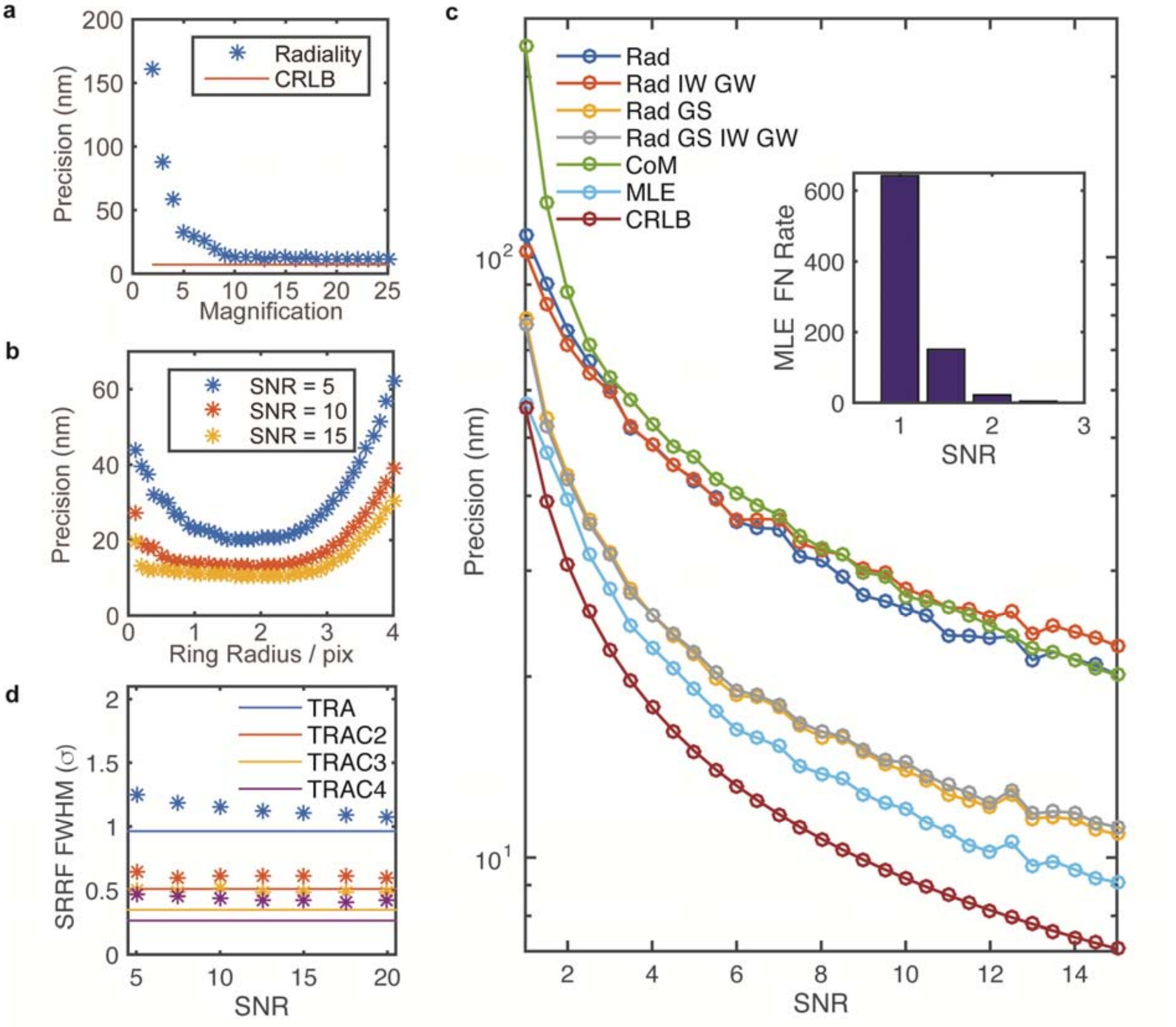

Supplementary Figure 3. Single fluorophore evaluation of SRRF. (a) Radiality precision at various magnifications compared to the Cramér-Rao lower bound (CRLB). **(b)** Radiality precision at various ring radius and SNR. **(c)** Comparison of the Radiality (Rad) precision including gradient smoothing (GS), intensity weighting (IW) and gradient weighting (GW) to centre of mass (CoM), maximum likelihood estimation (MLE) and the CRLB. (**Inset)** The false negative (FN) count for the MLE in (c). Precision refers to the root mean square error in the analysis of 1000 simulated single frame, single PSFs. **(d)** SRRF FWHM at varying SNR; analysed with different temporal methods. Solid lines represent the theoretical distributions. Stars represent the FWHM of radially averaged profiles of N=100, 100 frame simulated fluorophore fluctuation data sets.

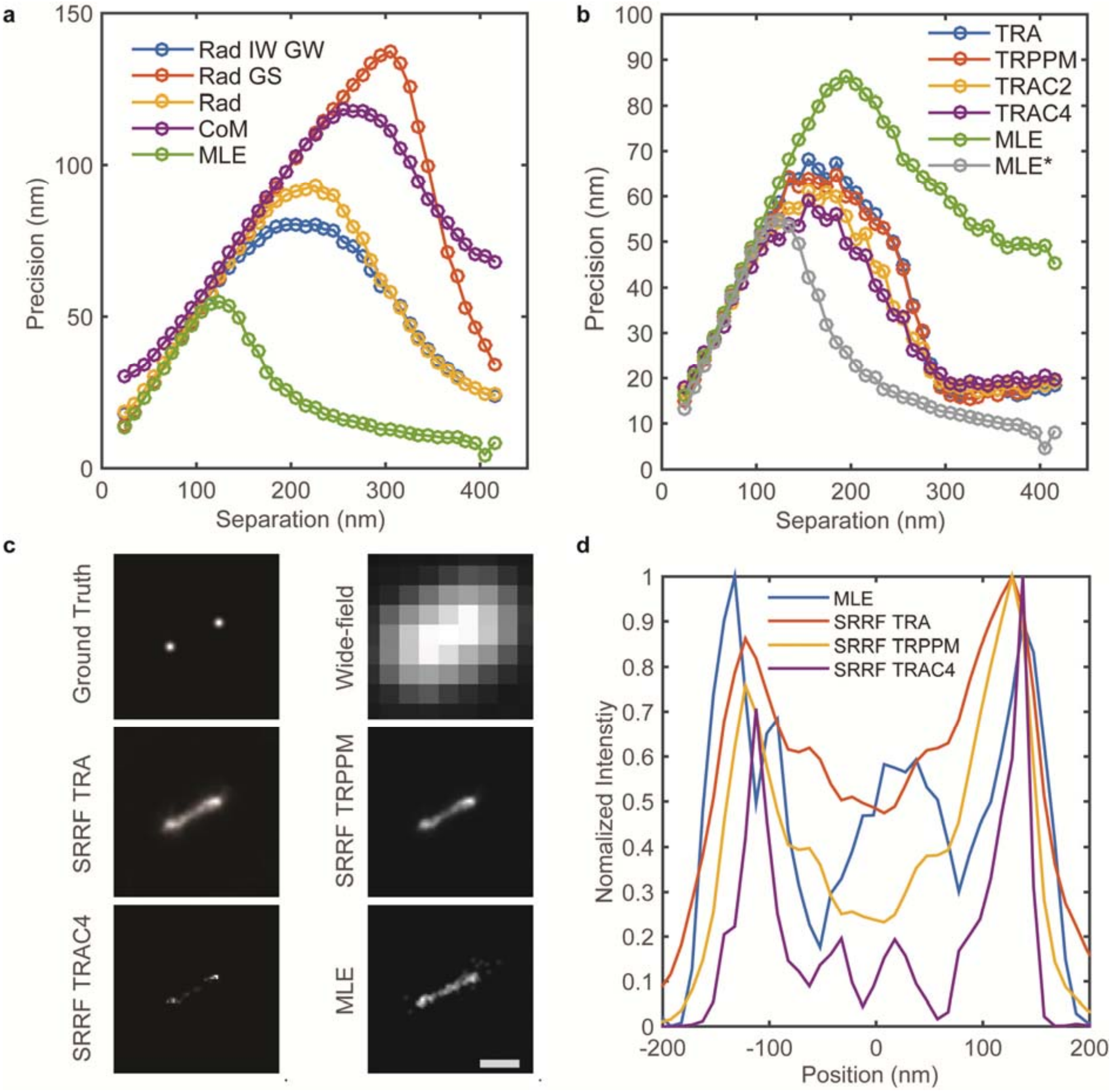

Supplementary Figure 4. Fluorophore pair evaluation of SRRF. (a) Comparison of the Radiality (Rad) precision for two emitters at various separations including gradient smoothing (GS), intensity weighting (IW) and gradient weighting (GW) to centre of mass (CoM) and multi-emitter maximum likelihood estimation (MLE). Precision refers to the root mean square error in the analysis of 1000 simulated single frame, emitter pairs without intensity fluctuations. **(b)** The SRRF precision for two emitters at various separations including temporal radiality average (TRA), pairwise product mean (TRPPM) and auto-cumulant order 2 and 4 (TRAC2,4) and multi-emitter maximum likelihood estimation (MLE). The multi-emitter maximum likelihood estimation precision without fluctuations from (a) (MLE**) is re-included for comparison in grey. Precision refers to the root mean square error in the analysis of 100, 100 frame simulations of fluctuating emitter pairs. **(c)** Comparison of representative (chosen randomly) 100 frame SRRF reconstruction for separation = 275 nm (0.85 times the FWHM) using TRA, TRPPM and TRAC4 to the MLE reconstruction, the ground truth and the widefield. Scale bar 200 nm **(d)** Line profiles through the known positions of the emitters for the reconstructions in (c).

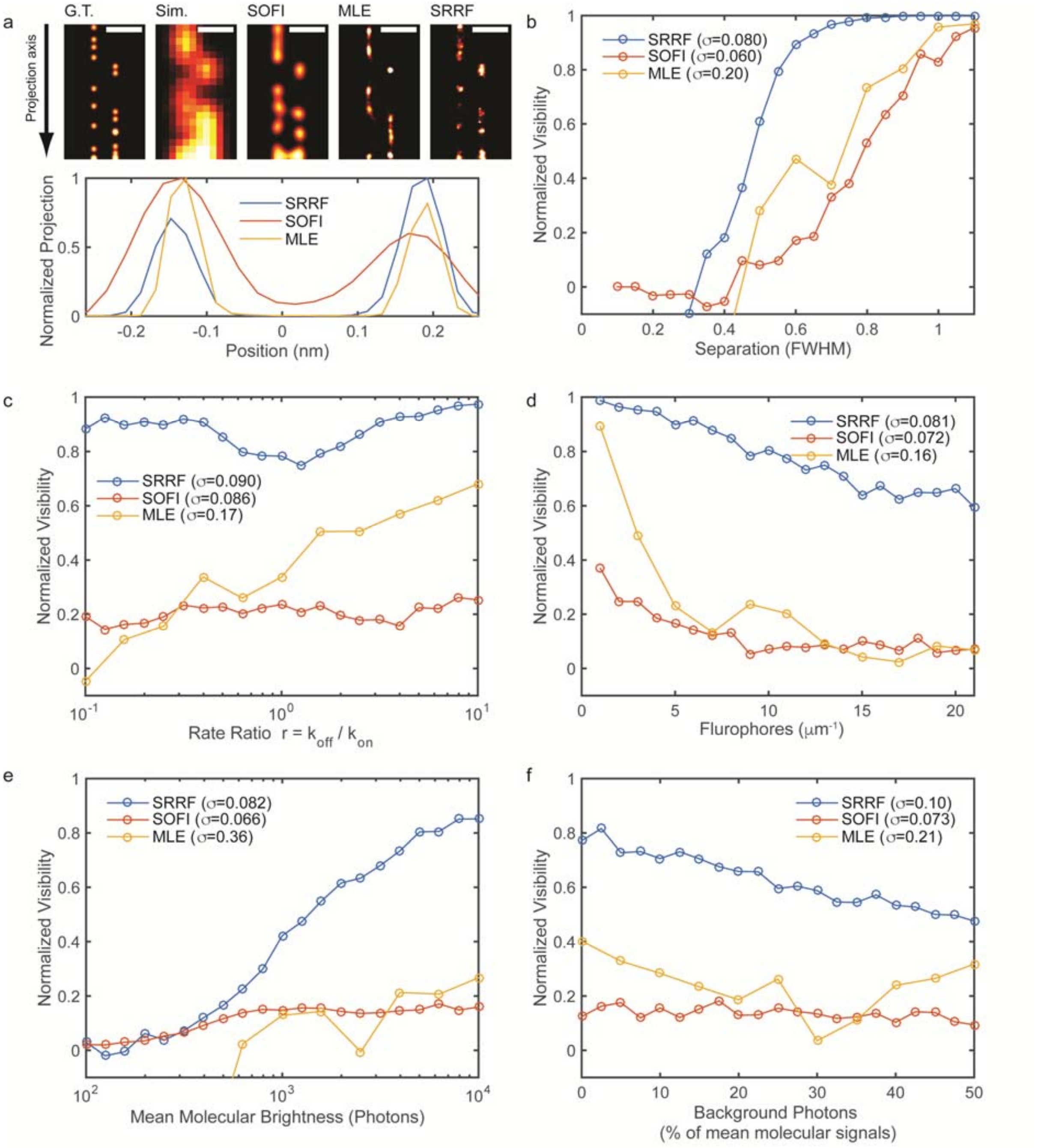

Supplementary Figure 5. Quantitative comparison of SRRF, SOFI and multi-emitter fitting by visibility analysis. (a) Top: Representative example (chosen randomly) of the ground truth (G.T.), a single simulation frame (Sim.), the SOFI reconstruction (SOFI), the multi-emitter maximum likelihood (MLE) reconstruction (MLE) and the SRRF reconstruction (SRRF) for separation = FWHM, rate ratio $r = k_{off} / k_{on}$ = 0.15, fluorophores μm^{-1} = 5, mean molecular brightness = 5000 photons s^{-1} and mean background photons = 100. Scale bar 500 nm. The projection axis used in the visibility analysis is indicated. **Bottom:** Normalized projected profiles from which the relative visibility is measured. Visibility analysis was performed for SOFI, multi-emitter maximum likelihood and SRRF as described in Methods. Parameters, **(b)** separation, **(c)** rate ratio, **(d)** labelling density, **(e)** mean molecular brightness and **(f)** background photons were varied independently from an otherwise fixed parameter set: separation = 0.6 x FWHM, rate ratio $r = k_{off} / k_{on}$ = 0.15, fluorophores μm^{-1} = 5, mean molecular brightness = 5000 photons s^{-1} and mean background photons = 2%. For all data points, SRRF N = 20, SOFI N = 20, multi-emitter maximum likelihood (MLE) N = 5 and the mean standard deviation for each method is indicated in the legend.

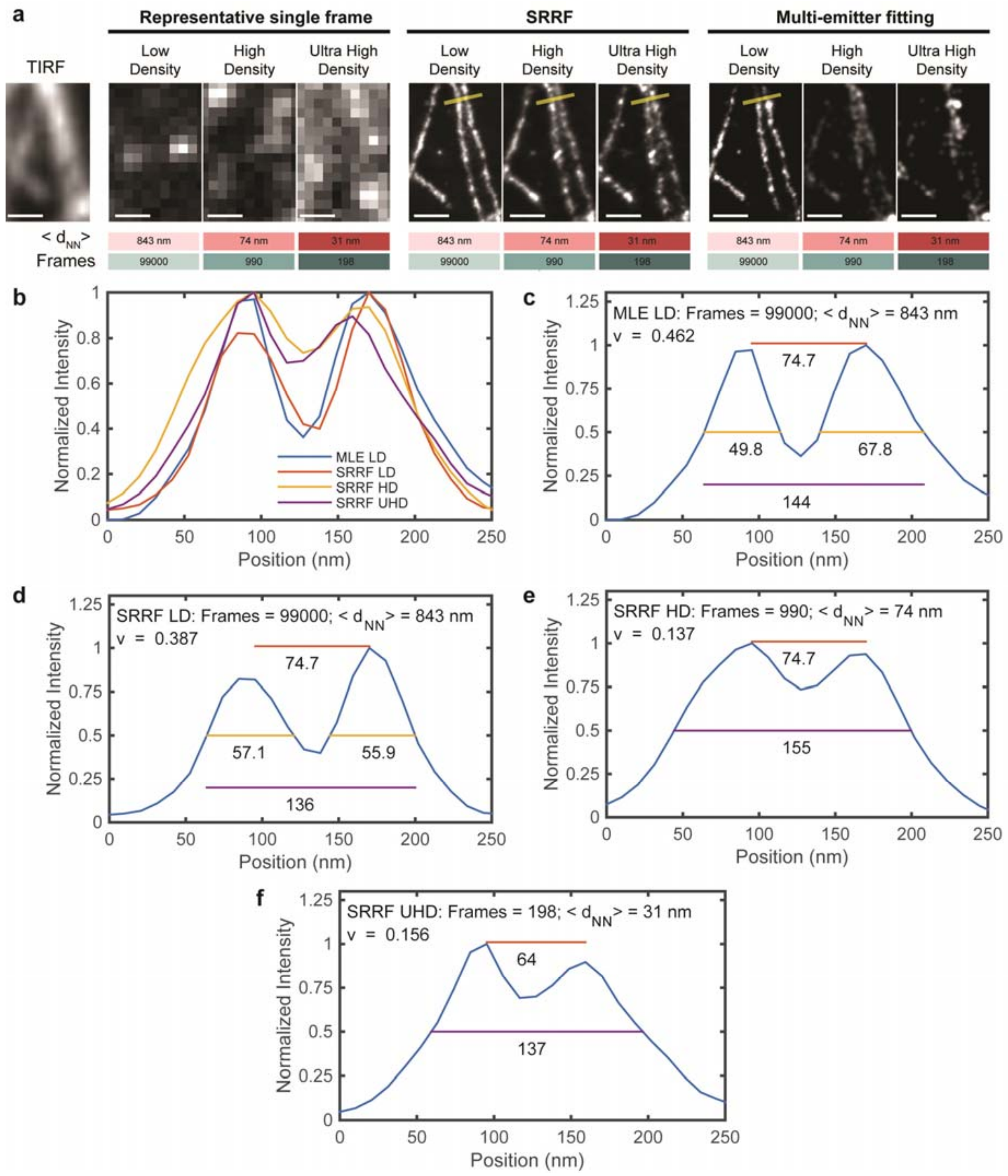

Supplementary Figure 6. Quantification of resolution at various densities using temporally averaged datasets. Analysis of resolution in high- and ultra-high density datasets created from temporally averaging a low density data set as described in Methods. **(a)** TIRF image of the structure for which the low density dataset was acquired, representative single frames from the three datasets (original low density and artificial high- and ultra-high density), and the corresponding SRRF and multi-emitter fitting reconstructions. The location from which plot profiles were measured, mean fluorophore separation per frame ($<d_{NN}>$) and number of frames in the dataset are indicated. Scale bar 500 nm. **(b)** Comparison of the plot profiles indicated in (a). Analysis of the peak to peak separation (red), full structure width (purple) and, where applicable, FWHM (yellow) for the **(c)** MLE low density and SRRF **(d)** low, **(e)** high and **(f)** ultra-high density datasets. The visibilities (v) calculated from these profiles are indicated.

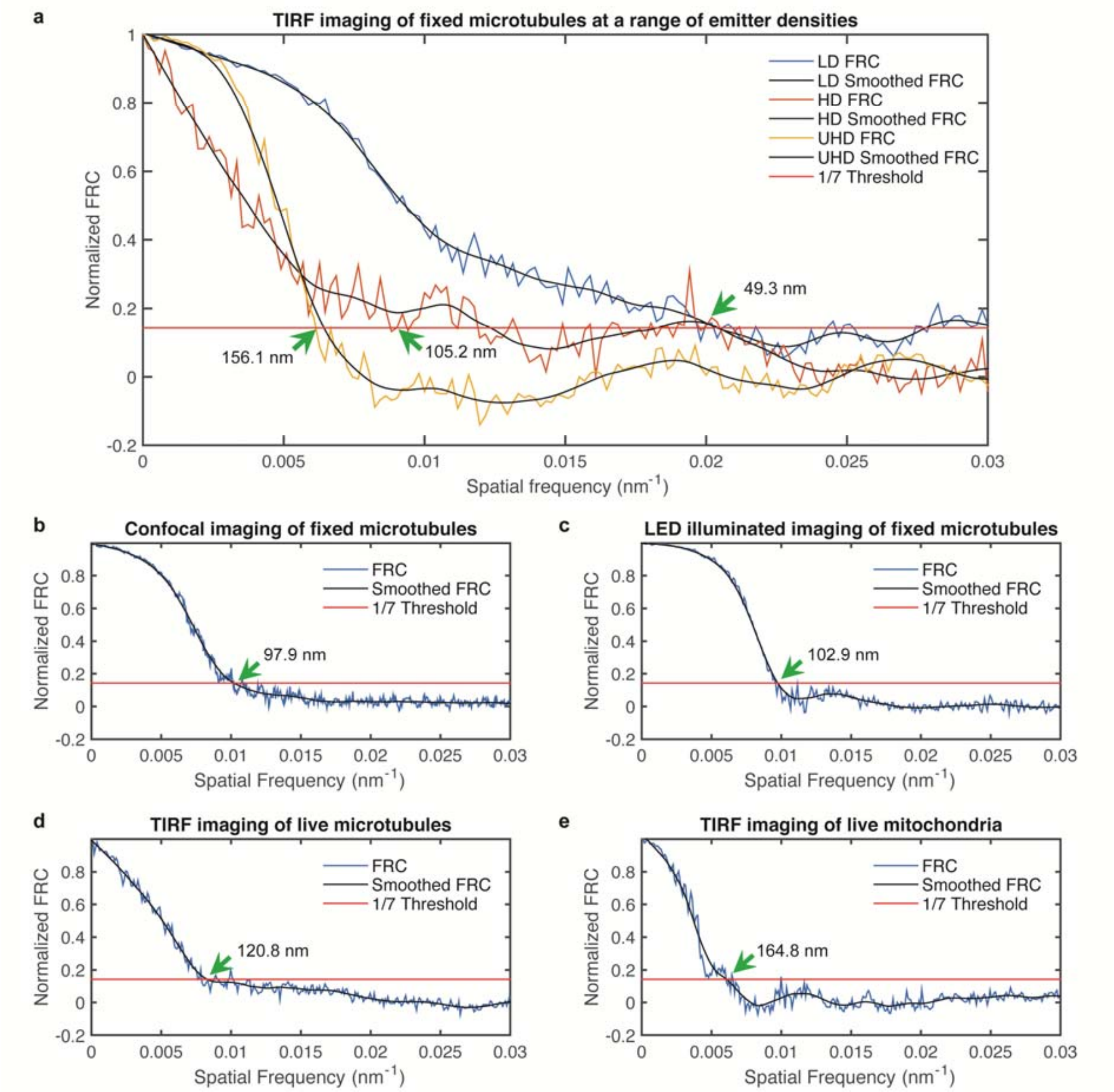

Supplementary Figure 7. Fourier Ring Correlation analysis of resolution. (a) Analysis of resolution by Fourier ring correlation (FRC) for low density (LD), high density (HD) and ultra-high density (UHD) data in Figure 2b. Analysis of resolution by Fourier ring correlation (FRC) for **(b)** confocal imaging and **(c)** LED illuminated widefield imaging of fixed microtubules (Fig. 3), **(d)** TIRF imaging of live microtubules (Supplementary Movie 2) and **(e)** TIRF imaging of live mitochondria (Supplementary Movie 4) respectively. Green arrows indicate the first crossing point of the 1/7 threshold shown used to determine the resolution in each case.

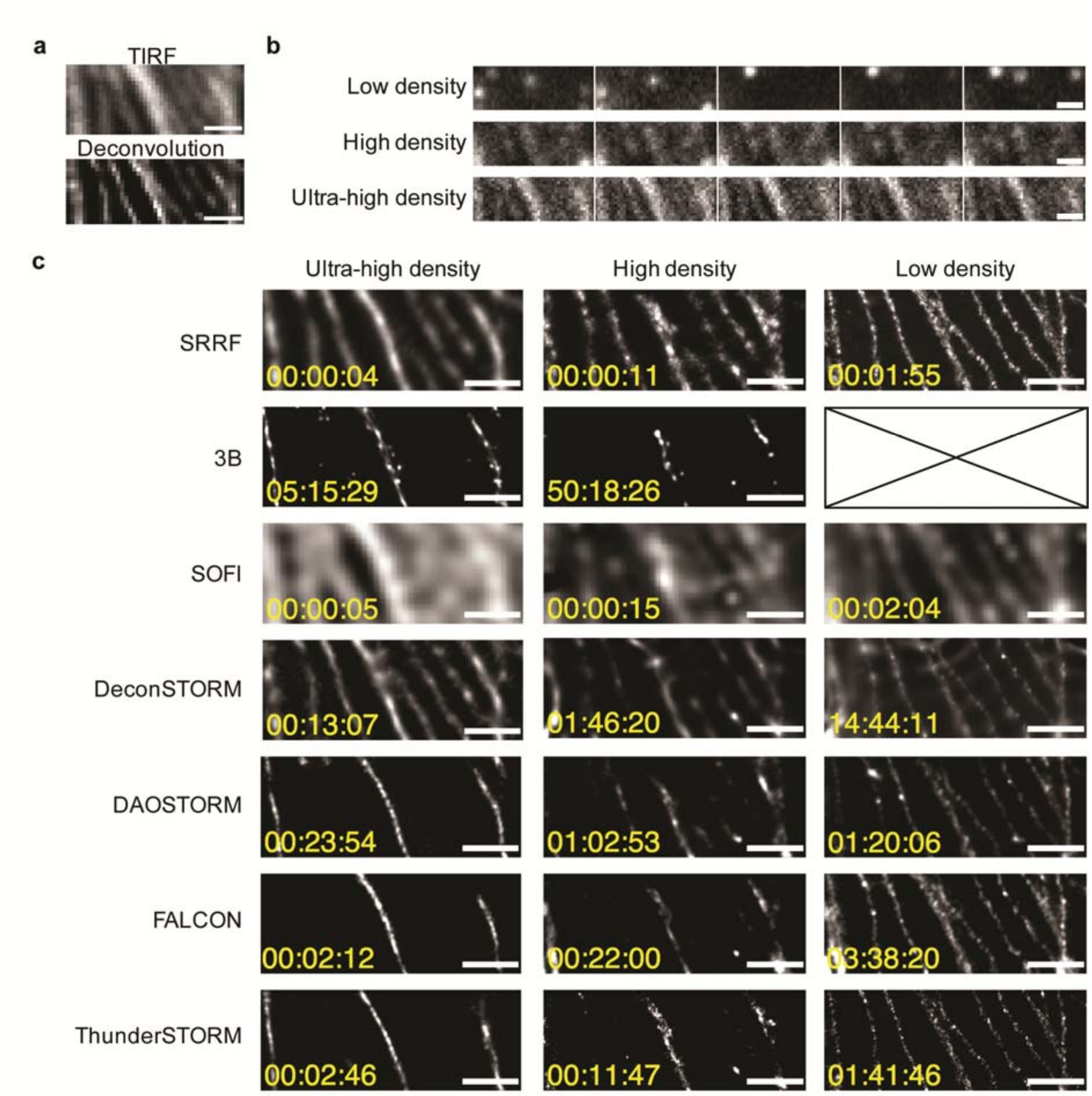

Supplementary Figure 8. Comparisons between SRRF and other super-resolution algorithms at a range of densities. (a) TIRF and deconvolved TIRF images of the data presented in Fig. 2b. **(b)** Five consecutive frames from the three different density datasets acquired for algorithm comparisons. Imaging parameters are as in Fig. 2b and Methods. **(c)** Images obtained using the listed algorithms to reconstruct the three different density datasets. The amount of time taken to produce each image is shown in yellow text in the format hh:mm:ss. 3B analysis was not performed on the low density data. Scalebars = 1 μm.

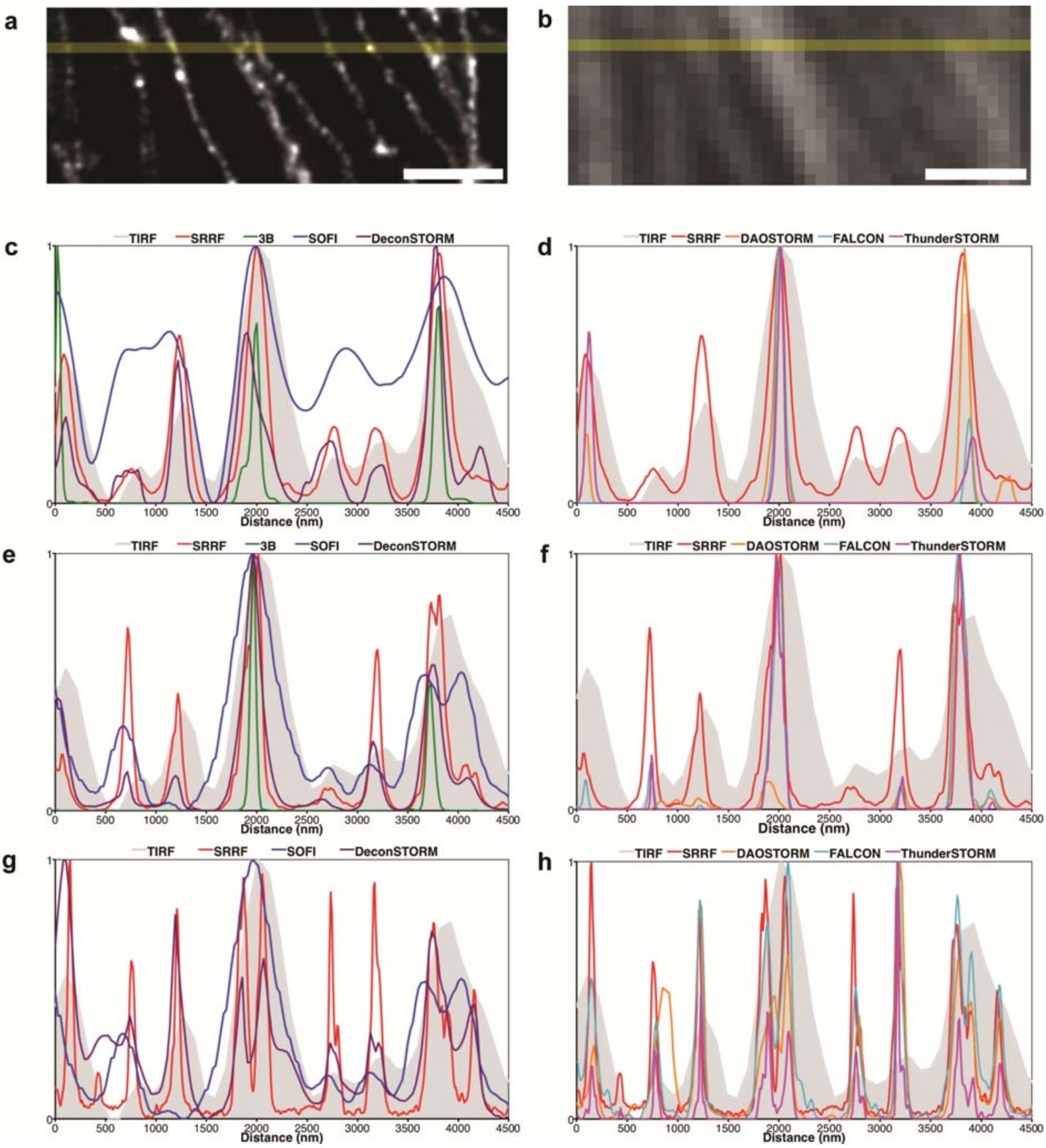

Supplementary Figure 9. Line profiles for super-resolution algorithm comparisons. The location at which line profiles were measured is shown as a yellow line on the **(a)** DAOSTORM low density reconstruction and **(b)** TIRF image. The height in the y direction of each profile was 100 nm, allowing for averaging over this range to prevent single pixel biases. Scalebars = 1 µm. Line profiles from the region indicated in (a) and (b) are plotted in the profiles below (intensities all normalized from 0 to 1). **(c-d)** Ultra-high density reconstruction profiles for SRRF compared with image-based techniques and localization techniques respectively. **(e-f)** High density reconstruction profiles for SRRF compared with image-based techniques and localization techniques respectively. **(g-h)** Low density reconstruction profiles for SRRF compared with image-based techniques and localization techniques.

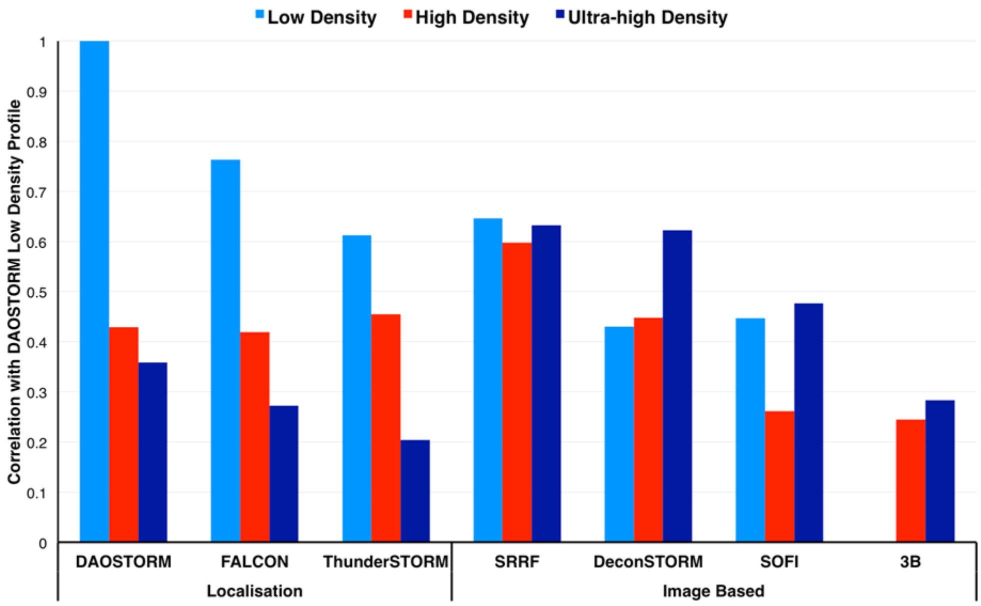

Supplementary Figure 10. Correlation scores for super-resolution algorithm comparisons. Plot showing values of Pearson's correlation coefficient for each line profile plotted in Fig. 9b-d when compared against the line profile for the low density DAOSTORM reconstruction (orange line, Fig. S9h)).

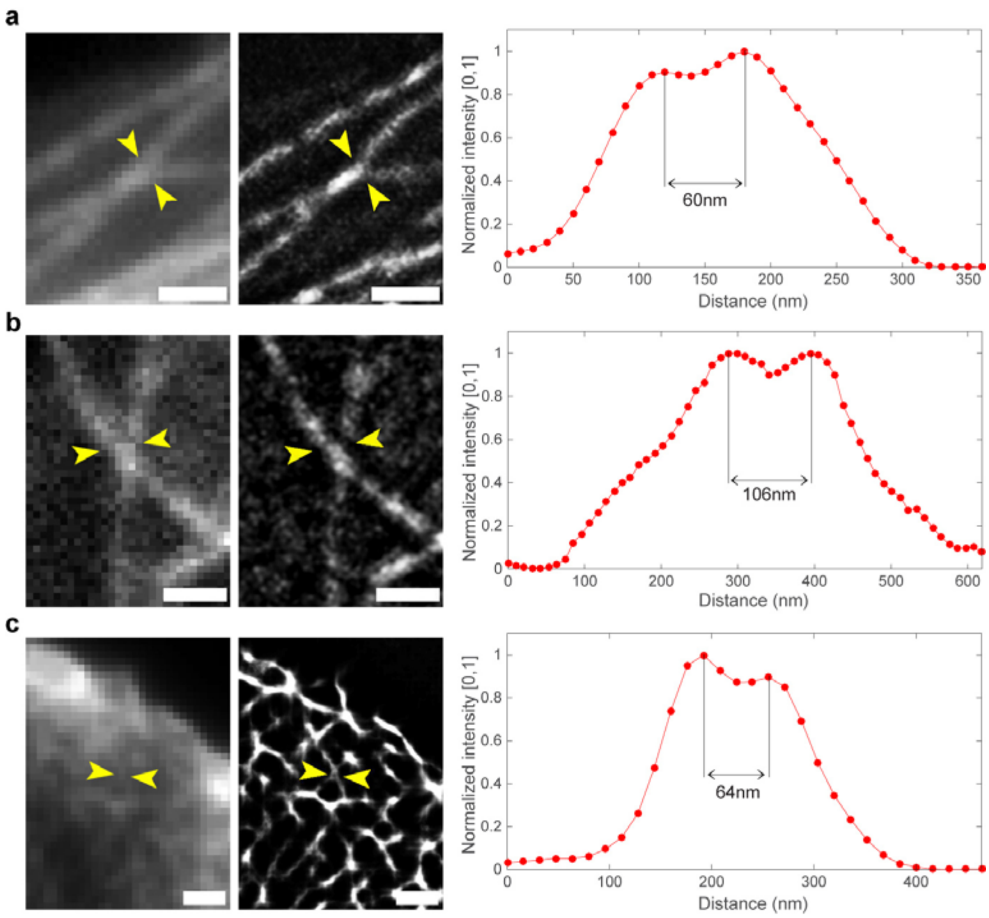

Supplementary Figure 11. Peak to peak separation of GFP-labelled structures in live cell movies. (a) Magnified view of two crossing microtubules from Supplementary Movie 2 and SRRF intensity plot taken between the yellow arrowheads. **(b)** Magnified view of two crossing microtubules from Supplementary Movie 3 and SRRF intensity plot taken between the yellow arrowheads. **(c)** Magnified view of two crossing actin structures from Supplementary Movie 8 and SRRF intensity plot taken between the yellow arrowheads. All scalebars = 1 µm. Line profiles were averaged over ~100 nm adjacent to the line to ensure profiles were not corrupted by noise peaks.

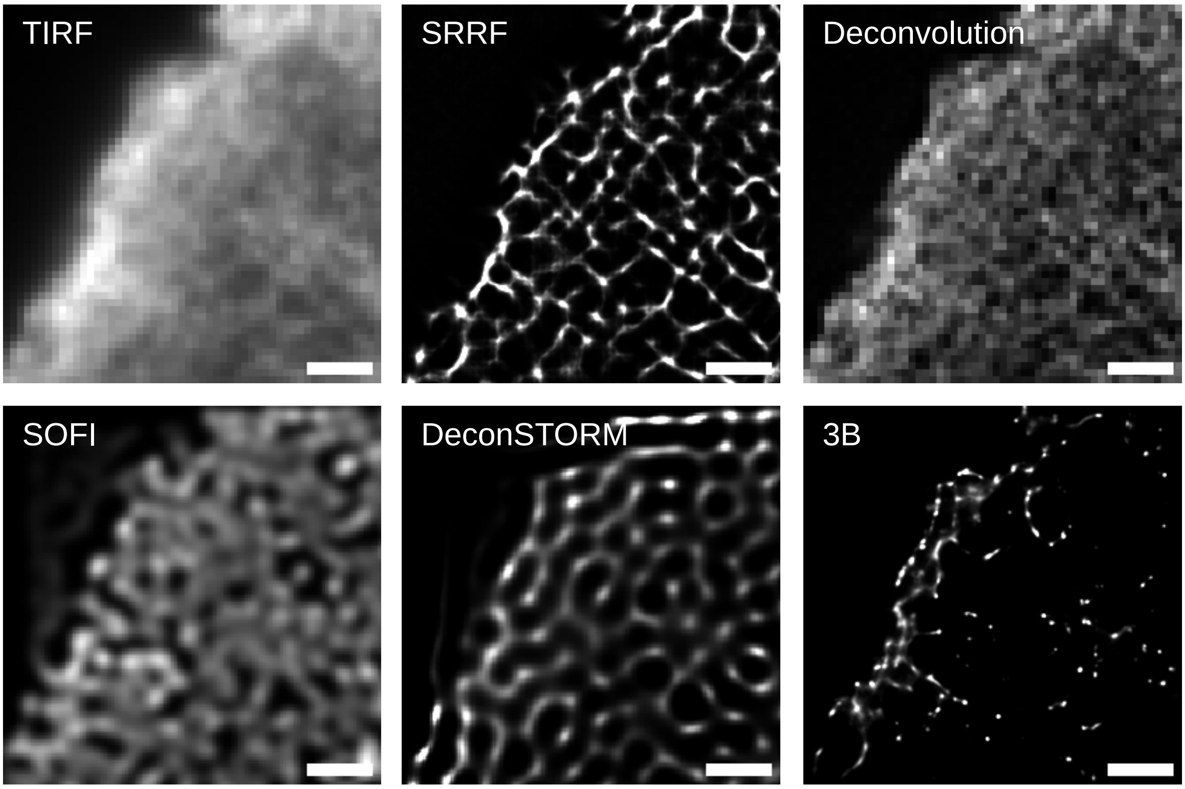

Supplementary Figure 12. Actin organisation in a live activated T-cell reconstructed using high density algorithms. Region of 100 raw frames of LifeAct-GFP in the T-cell stimulated with anti-CD3&CD28 as shown in Fig. 4b and Supplementary Movie 8 reconstructed with the various image-based techniques. Scalebars = 1 µm.

**Dataset**	**On-sample intensity (kW cm^{-2})**	**Total imaging time (s)**
Alexa Fluor 647 labelled microtubules in fixed CHO cells	4	1800
Alexa Fluor 647 labelled microtubules in fixed LLC-MK2 cells	2.31	1042
LifeAct-GFP Jurkat T-cells stimulated with anti-CD28	0.21	50
LifeAct-GFP Jurkat T-cells stimulated with anti-CD3&CD28	0.083	50
LifeAct-GFP Jurkat T-cells stimulated with anti-CD3	0.083	50
Tubulin-GFP HeLa cells – long term imaging every 25 mins for 8 hours	0.065	20
LifeAct-GFP Jurkat T-cell drop onto anti-CD3	0.042	200
Tubulin-GFP HeLa cells – short term continuous imaging	0.0085	51
Mitotracker red HeLa cells	0.00025	121

Supplementary Table 1. Laser intensities and total imaging time for datasets displayed. Summary of the on-sample intensities (kW cm^{-2}) and the total imaging time (s) for the data sets included in this paper.

**Parameter**	**Description** (further details available in Supplementary Note 1 and Supplementary Software Manual)
Ring Radius	Sets the radius (in pixels) at which gradient convergence is calculated. Determines the FWHM and the precision of the Radiality distribution. See Supplementary Figs. 1 and 3b.
Radiality Magnification	Sets the factor decrease in the 1D sub pixel size relative to the original pixel size. Determines the spatial support of the SRRF image and affects the algorithm run time. See Supplementary Fig. 3a.
Axes in Ring	Sets the number of symmetry axes to be used in the Radiality calculation. N 'ring coordinates' (Supplementary Note 1) is double the number of symmetry axes. Determines the sampling frequency of the local gradient field and affects the algorithm run time.
Remove Positivity Constraint	Allows Radiality distribution to have negative values.
Renormalize	Renormalizes the Radiality distribution range from [-1 1] to [0 1].
Do Gradient Smoothing	Applies a larger 5x3 and 3x5 first derivative kernel pair to gradient calculation. Increases accuracy of Radiality distribution at low SNR but decreases sensitivity to high spatial frequencies. See Supplementary Figs. 3c and 4a.
Do Intensity Weighting	Weights the Radiality distribution by intensity. Increases contrast and SNR of the radiality distribution. See Supplementary Figs. 3c and 4a.
Do Gradient Weighting	Weights the Radiality distribution by local gradient magnitude. Increases contrast and SNR of the Radiality distribution. See Supplementary Figs. 3c and 4a.
PSF FWHM	Determines the Ring Radius of the local gradient magnitude calculation; only applies to Gradient Weighting.
Temporal Analysis Method	Chooses between temporal analysis methods, TRM, TRA, TRPPM and TRAC. See Supplementary Figs. 1d, 3d and 4.
Integrate Temporal Correlations	Calculates the cumulant integrated over all time lags (TRAC only).
TRAC Order	Sets the order of the cumulant analysis (TRAC only). See Supplementary Figs. 1d, 3d and 4.
Do Temporal Subtraction	Applies a frame by frame subtraction to the raw data before calculation of SRRF (as described by Burnette et al[S1]).

Supplementary Table 2. Summary of user defined parameters in the SRRF algorithm. User defined parameters in the SRRF algorithm are summarised with reference to figures and text providing additional details.

Supplementary Note 1: Super-Resolution Radial Fluctuations Algorithm

The Super-Resolution Radial Fluctuations (SRRF) algorithm is comprised of two distinct parts, a spatial and a temporal analysis, that when combined efficiently super-resolve cellular structures from a temporal image sequence of fluorophores oscillating their emission (Fig. 1).

The first part, spatial analysis, corresponds to the calculation of the degree of radial symmetry for sub-pixel regions. This procedure applied to an image generates what we refer to here as a radiality map. Each frame on the original image sequence will be analysed by this procedure, generating a corresponding sequence of radiality maps. The second step, temporal analysis, entails the analysis of the sequence of radiality maps through higher-order temporal statistics, such as cumulant analysis, generating a single super-resolution image referred to here as a SRRF map.

Spatial Analysis – Generating Radiality Maps

We quantify the radial symmetry of a point in space by measuring the extent to which the local image gradients converge on this point (Fig. 1a). Measuring the average distance to lines of gradient around a point in an image provides a measure of the degree of convergence of the gradient lines. Thus, the radiality map of an image is calculated as follows.

For an image plane $I(x, y)$, the gradients $\mathbf{G}_\mathrm{x}$ and $\mathbf{G}_\mathrm{y}$ can be calculated separately as per Equation S1.

$$\mathbf{G}_\mathrm{x}(x,y) = \frac{\partial I(x,y)}{\partial x} \qquad S1$$
$$\mathbf{G}_\mathrm{y}(x,y) = \frac{\partial I(x,y)}{\partial y}$$

For every pixel in the original image, $I(x, y)$, a good first order approximation to the image gradients in x and y directions, $\mathbf{G}_\mathrm{x}$ and $\mathbf{G}_\mathrm{y}$ can be calculated, without translation, by convolution with a differentiation kernel pair[S2] (Equations S2,3).

$$\mathbf{G}_\mathrm{x} = [-1 \quad 0 \quad 1] * I \qquad S2$$
$$\mathbf{G}_\mathrm{y} = \begin{bmatrix} -1 \\ 0 \\ 1 \end{bmatrix} * I$$

In low densities this kernel pair can be increased to a 5x3 and 3x5 first derivative kernel pair (Equation S3) in order to be more robust to low SNR however sensitivity to the higher spatial frequencies in the image is compromised by the spatial averaging of the gradient fields.

$$\mathbf{G}_\mathrm{x} = \begin{bmatrix} -1 & -1 & 0 & 1 & 1 \\ -1 & -1 & 0 & 1 & 1 \\ -1 & -1 & 0 & 1 & 1 \end{bmatrix} * I \qquad S3$$
$$\mathbf{G}_\mathrm{y} = \begin{bmatrix} -1 & -1 & -1 \\ -1 & -1 & -1 \\ 0 & 0 & 0 \\ 1 & 1 & 1 \\ 1 & 1 & 1 \end{bmatrix} * I$$

From this point calculations are performed in continuous space in order to populate a magnified analysis grid. As such we define a set of locations (x_c, y_c) in a magnified pixel grid for which the calculation of radiality is performed (Supplementary Fig. 1a). Note that in general these points will be the centre of each magnified pixel. If a drift correction table is provided however, the drift can be corrected for by shifting the point (x_c, y_c) by the corresponding drift vector for the current frame (purple line Supplementary Fig. 1a). This shift can be performed in continuous space without altering the subsequent calculations in any way. A drift correction table can be provided by the user or

calculated with the normalised cross correlation based drift estimation method[S3,S4,S5] included in the NanoJ-SRRF plugin.

From the point (x_c, y_c), N 'ring co-ordinates' (x'_i, y'_i) are defined such that they are equally distributed around a ring of radius r (Supplementary Fig. 1a). N is an arbitrarily defined variable that determines the number of gradient samples, in this algorithm it takes a value of 12 by default. At each of these co-ordinates in continuous space the gradients G_{xi} and G_{yi} are calculated by Catmull-Rom spline interpolation[S6] from the original image gradients $\mathbf{G}_\mathrm{x}$ and $\mathbf{G}_\mathrm{y}$. The equation of a line describing this gradient can be formulated (Equation S4) and in general the perpendicular distance from a point (X, Y) to a line is given by Equation S5.

$$0 = (x - x'_i)G_{yi} - (y - y'_i)G_{xi} \quad \text{S4}$$

$$\mathrm{Dist}\big(ax + by + c = 0; (X, Y)\big) = \frac{|aX + bY + c|}{\sqrt{a^2+b^2}} \quad \text{S5}$$

This allows the calculation of the minimum perpendicular distance (Supplementary Fig. 1b), d_i, from the point (x_c, y_c) to the gradient line through the point (x'_i, y'_i) to be calculated as follows,

$$d_i = \frac{|(x_c - x'_i)G_{yi} - (y_c - y'_i)G_{xi}|}{\sqrt{{G_{xi}}^2 + {G_{yi}}^2}}. \quad \text{S6}$$

The normalised dot product, Θ, between the vectors $\mathbf{G}_\mathrm{i} = (G_{xi}\hat{\mathbf{x}}, G_{yi}\hat{\mathbf{y}})$ and $\mathbf{r}_\mathrm{i} = (r_{xi}\hat{\mathbf{x}}, r_{yi}\hat{\mathbf{y}})$ can be calculated,

$$\Theta = \frac{\mathbf{G}_\mathrm{i} . \mathbf{r}_\mathrm{i}}{|\mathbf{G}_\mathrm{i}||\mathbf{r}_\mathrm{i}|}, \quad \text{S7}$$

which is positive if the gradient is convergent and negative if the gradient is divergent. We then define the degree of convergence of the gradient line on the point (x_c, y_c) as

$$c_i = \mathrm{sgn}(\Theta)\left[\frac{(1 - d_i)}{|\mathbf{r}_\mathrm{i}|}\right]^2, \quad \text{S8}$$

that can take values from -1 to 1 and is dependent on the angle θ alone (Supplementary Fig. 1c). The mean convergence for the N ring co-ordinates is calculated to give the raw un-weighted radiality in the pixel (x, y) in frame t,

$$R_t(x, y) = \frac{1}{N}\sum_{i=1}^{N} c_i. \quad \text{S9}$$

The raw radiality, $R_t(x, y)$, can subsequently be weighted by the interpolated intensity at the point (x_c, y_c). Alternatively or in addition, the radiality can be weighted by a sum of the local gradient magnitudes. A second ring with an independently chosen radius is used to determine the gradient weight, G_w, calculated as

$$G_\mathrm{w} = \frac{1}{N}\sum_{i=1}^{N} \frac{\mathrm{sgn}(\Theta)(|\mathbf{G}_\mathrm{i}| - |\mathbf{G}_\mathrm{c}|)}{I_c}, \quad \text{S10}$$

where Θ and $\mathbf{G}_\mathrm{i}$ are recalculated for the new ring co-ordinates and $\mathbf{G}_\mathrm{c}$ and I_c are the gradient and intensities respectively at the point (x_c, y_c). Regions of the resulting radiality map can be negative, indicating divergence in the gradient field and suggesting the absence of an emitter at these locations.

Since the absence of an emitter in a single frame does not diminish the probability of an emitter appearing in subsequent frames, negative radialities are set to zero. This positivity constraint can be disabled in the NanoJ-SRRF plugin. At this stage, the presence of radially symmetric PSF profiles caused by fluorophores in an image will lead to equivalent conically shaped peaks appearing on the corresponding radiality map, with a FWHM proportional to the ring radius used (Supplementary Fig. 1d). We solved Equation S9 numerically (Mathematica, Wolfram Research, Inc) demonstrating that two, equally intense Gaussians, separated by <0.7 times their FWHM can be resolved in a single time point (frame) by calculating the radiality map. Supplementary Movie 1 demonstrates the effect on the radiality map of varying key parameters such as ring radius and PSF size, shape and position.

Temporal Analysis – Generating the SRRF Map

Following calculation of the radiality image sequence a single super-resolution frame can be generated from a pixel-by-pixel maximum projection, temporal radiality maximum (TRM), or average projection, temporal radiality average (TRA). In datasets where peak separation is greater than 0.7 times the FWHM of the PSF, emitters can be directly resolved by the radiality map without further enhancement brought by higher-order temporal correlations. Thus, TRM and TRA projections of the radiality image sequence will produce super-resolution reconstructions with resolutions dependent on the chosen ring radius and the SNR (Supplementary Figs. 1, 3). Each method has an advantage as the TRA will be less influenced by noise whereas TRM will have the benefit of not being dominated by constantly emitting sources such as fiducial markers. If persistently emitting sources are present in the data however, these can also be removed by frame-by-frame temporal subtraction[S1] prior to the calculation of the radiality.

Further resolution gains and significant improvements in fidelity and contrast can be achieved however, if the temporal fluctuations in the radiality maps are analysed using higher-order statistics.

The NanoJ-SRRF plugin offers four options for higher-order temporal analysis. The first, temporal radiality pairwise product mean, TRPPM, calculates the second order raw-moment, integrated over all time lags and normalised by the number of frame pairs used. This can be formulated as follows,

$$\mathrm{TRPPM}(x,y) = \frac{2}{T(1+T)} \sum_{s=0}^{T-1} \sum_{t=s}^{T-1} R_s R_t, \qquad S11$$

where T is the total number of frames, s and t enumerate the frames and R_s and R_t are the radialities at the pixel (x, y) in frame s and t respectively.

The other three methods, temporal radiality auto-cumulant, TRAC 2, 3 and 4, constitute the second, third and fourth order central auto-cumulants with a time lag, δt, of 1 frame as previously discussed in Dertinger, T. et al[S7] and formulated here, as follows,

$$\mathrm{TRAC2}(x,y) = \langle \delta R_t \cdot \delta R_{t+\delta t} \rangle, \qquad S12$$

$$\mathrm{TRAC3}(x,y) = \langle \delta R_t \cdot \delta R_{t+\delta t} \cdot \delta R_{t+2\delta t} \rangle,$$

$$\begin{aligned}\mathrm{TRAC4}(x,y) = &\langle \delta R_t \cdot \delta R_{t+\delta t} \cdot \delta R_{t+2\delta t} \cdot \delta R_{t+3\delta t} \rangle \\ &- \langle \delta R_t \cdot \delta R_{t+\delta t} \rangle \langle \delta R_{t+2\delta t} \cdot \delta R_{t+3\delta t} \rangle \\ &- \langle \delta R_t \cdot \delta R_{t+2\delta t} \rangle \langle \delta R_{t+\delta t} \cdot \delta R_{t+3\delta t} \rangle \\ &- \langle \delta R_t \cdot \delta R_{t+3\delta t} \rangle \langle \delta R_{t+\delta t} \cdot \delta R_{t+2\delta t} \rangle\end{aligned}$$

where $\delta R_t = R_t - \langle R \rangle$, and $\langle \cdots \rangle$ indicates an average over time. For a comprehensive overview of cumulant analysis see Mendel, J.M.[S8].

Note on the implementation of the ImageJ SRRF plugin

The NanoJ-SRRF ImageJ plugin (Supplementary Software) provided includes a graphical user interface allowing options for data management, time-lapse analysis, drift correction and temporal subtraction as well as the user defined algorithm parameters. These user defined parameters are summarised in Supplementary Table 2. A software manual (Supplementary Software) is also provided describing installation of the plugin and giving further information on the selection of algorithm parameters.

The calculation of the radiality of each sub-pixel is trivially parallelisable since each calculation is independent of all other sub pixels in time and space. Similarly, the temporal calculation for each sub-pixel is independent of all other sub pixels in space and can be parallelised with respect to space. A computationally efficient approximate calculation of the auto cumulant integrated over all time lags excluding $\delta t = 0$ is also implemented using a course-grained binning approach adopted from fluorescence correlation spectroscopy[S9]. NanoJ-SRRF performs these calculations on a graphical processing card, where one is available, allowing massive parallelisation enabling a typical 512x512 pixel data set to be analysed at 1 frame per second on a 2013 2.4GHz Intel i7-4700MQ notebook processor with integrated graphics card (Supplementary Fig. 1e). A flow-chart outlining the main elements of SRRF is presented in Supplementary Fig. 2.

The NanoJ-SRRF plugin additionally provides the functionality to test its environment for suitable CPU and GPU hardware allowing the user to select the most suitable environment. Debugging options are also provided.

Note on temporal analysis of radiality

Dertinger et al[S7], in the formulation of the SOFI algorithm, make the assumption that the fluorescence distribution arises from a set of fluorophores with statistically independent intensity fluctuations in stationary equilibrium. This allows, as in Equation 3 of Dertinger et al, the fluorescence, $F(\mathbf{r}, t)$ at position $\mathbf{r}$, time t to be expressed as a sum of the zero mean fluctuations of N fluorophores,

$$\delta F(\mathbf{r}, t) = \sum_{k}^{N} U(\mathbf{r} - \mathbf{r}_k) \cdot \varepsilon_k \cdot \delta s_k(t), \tag{S13}$$

where $U(\mathbf{r} - \mathbf{r}_k) \cdot \varepsilon_k \cdot \delta s_k(t)$ is the convolution of the system PSF, $U(\mathbf{r})$, and the kth contribution to the fluorescence source distribution having mean brightness, ε_k, and time dependent fluctuation $\delta s_k(t)$. Applying a second order auto correlation function to the fluorescence fluctuations (Equation 4 Dertinger et al) yields

$$G_2(\mathbf{r}, t) = \sum_{j,k}^{N} U(\mathbf{r} - \mathbf{r}_j) U(\mathbf{r} - \mathbf{r}_k) \cdot \varepsilon_j \cdot \varepsilon_k \cdot \langle \delta s_j(t + \tau) \delta s_k(t) \rangle, \tag{S14}$$

where $\langle \dots \rangle$ denotes a time average. When the fluorescence intensity fluctuations are transformed to radiality fluctuations however a dependence on the local environment of the image is introduced into the correlation function. This dependence prevents the simple reduction of the correlation function via the assumptions made by Dertinger et al.

In the case of very low densities where no fluorophores overlap in space within a pair of frames then there are no cross correlation terms in the autocorrelation function and the radiality intensity $\varepsilon_k \approx 1$. It has been shown previously that the nth order auto-cumulant analysis of a fluctuating Gaussian intensity distribution bestows a factor of $\sqrt{n}$ decrease in the FWHM of the PSF since the extent of correlation varies non-linearly across the distribution[S7]. We empirically demonstrate a similar FWHM decrease in the non-Gaussian radiality PSF using TRPPM and TRAC 2, 3 and 4 applied to a

simulated, single fluorophore (Supplementary Fig. 1d). Under this best-case scenario the resulting SRRF intensity distribution is equal to the radiality PSF raised to the power 2, 3 or 4 depending on the auto-cumulant order and weighted by the molecular correlation function. This is demonstrated by simulation of the ideal case of a single fluctuating fluorophore with no noise (Supplementary Fig. 1d) and is shown to agree well with the theoretical model of the radiality PSF squared. Good agreement is also seen with the theoretical distribution for 3rd and 4th order auto-cumulants.

In the case of multiple fluorophores coexisting in the same spatial and temporal volume however, the appearance of an additional fluorophore will reduce the radiality of adjacent fluorophores while the disappearance of a fluorophore will increase the radiality of adjacent fluorophores (Supplementary Movie 1). This results in a radiality brightness $\varepsilon_k \neq 1$ reintroducing some of the non-linear brightness effects seen in SOFI. Since the appearance and disappearance of fluorophores can still be assumed to be independent, the cross-correlation terms in the auto-correlation function should still in general be approximately equal to zero despite the intensity distribution not being a simple sum of fluorophore intensities. We demonstrate in Supplementary Fig. 4d that the radiality PSF FWHM is in fact reduced when higher-order cumulants are applied to the radiality of adjacent fluorophores however as anticipated the effect is less pronounced than what might be expected in the single molecule case. The application of higher-order statistical analysis to pairs of fluctuating fluorophores however, demonstrates a notable improvement in the ability to resolve fluorophores at smaller separations (Supplementary Fig. 4b). Moreover, since the radiality peaks that occur as a result of noise are always uncorrelated in time it will in general be useful to use higher-order statistics in order to de-noise and enhance the contrast of the SRRF reconstructions.

Supplementary Note 2: Characterisation of performance across regimes

The evaluation of localisation algorithms has typically been based on the comparison of localisations made on simulated datasets, to the true simulated positions. The minimum theoretically achievable mean square error in localisation is given by the Cramér-Rao lower bound (CRLB) which can be approximated by Equation 7 from Rieger, B. & Stallinga, S.[S10],

$$\Delta x|^2_{\mathrm{MLE}} = \frac{\sigma^2 + a^2/12}{N}\left(1 + 4\tau + \sqrt{\frac{2\tau}{1+4\tau}}\right), \qquad S15$$

where N is the number of photons, σ is the PSF width, a is the pixel size and τ approximates the ratio of the background intensity to the peak signal intensity.

Since SRRF is a non-localisation based algorithm that produces single super-resolution images, a table of localised positions is not available for comparison with the ground truth. Instead, in order to provide a comparison to the CRLB, we have used single frame simulations of single fluorophores at various signal to noise ratios and compared the location of the pixel with the maximum radiality to the ground truth location. Due to the discrete nature of the possible locations of the radiality maxima, this method gives an approximation to the true precision of the radiality peak. This approximation is dependent on the magnification of the sub-pixel grid on which the radiality calculation is performed (Supplementary Fig. 3a). If the magnification is such that the sub-pixel size is smaller than the true precision the best possible estimate of the precision is achieved which is seen to approach the CRLB.

We first tested the precision of radiality as the ring radius, r in Supplementary Fig. 1, was varied. The ring radius is an important parameter as it determines the proximity of the gradient lines to the point of interest. As a consequence, the ring radius determines the maximum spatial frequency of the radiality map by changing the full width half maximum (FWHM) of the of the resulting radiality distribution. The precision of the radiality calculation will be influenced however, by the magnitude of the gradient from which it is calculated. This results in a reduction of the precision at small ring radius where the gradient at the centre of the emitter intensity distribution approaches zero (Supplementary Fig. 3b).

A principal determinant of the precision of localisation microscopy, as can be deduced from the equation for the CRLB, is the signal to noise ratio (SNR). We determined the precision of radiality at a range of SNRs from 1, equivalent to ~90 photons per emitter, to 15, equivalent ~1300 photons per emitter (Supplementary Fig. 3c). A selection of possible weighting methods and gradient kernels were tested. These results indicate that intensity weighting (IW) and gradient weighting (GW) both have a very small effect on the precision of radiality. However, the use of the larger (3x5 and 5x3) gradient smoothing (GS) kernels (Equation S3) has a marked increase in precision. For comparison, the precision of centre of mass (CoM) estimation[S11] and maximum likelihood estimation (MLE)[S3] were also calculated for the same datasets. In the case of the MLE the inset in Supplementary Fig. 3c shows the false negative (FN) count, the number of frames in which no fitting was performed as the algorithm was unable to detect a suitable candidate signal to fit the model to.

Aside from the interdependence between the radialities of adjacent fluorophores another important factor in determining the final FWHM of the SRRF reconstruction is the SNR. This is because despite the highly accurate peak position the displacement of the peak due to noise can approach a similar scale to the FWHM of the radiality peak (before temporal analysis). Temporal averaging or correlation of this displacement will result in a broadening of the SRRF FWHM while increasing the precision of the peak position. This effect is demonstrated for a fluctuating fluorophore in Supplementary Fig. 3d.

Many localisation algorithms achieve close to CRLB precision on single emitter localisations but suffer from a significant loss of precision when localising pairs of emitters separated by less than $\sim 3\sigma$, where σ, the standard deviation of a Gaussian PSF, is approximately 135 nm for wavelengths corresponding to Alexa Fluor 647 for example. The performance of radiality in the case of nearby emitters was investigated by simulating two equal intensity emitters at various separations (Supplementary Fig. 4a). The error in this case was calculated as the distance to the nearest pixel that was a local maximum. Again, error estimates from CoM and MLE (using a multi-emitter model) are included for comparison, for which the errors were calculated as the distance from each true position to the nearest localisation. The linear response initially at low separations indicates the condition where a single radiality peak is observed on the line between the two emitters. Any precision estimates lower than this line, equal to half the separation, indicates separation into two distinct peaks. These results indicate that GW and IW improve the precision of radiality when emitters overlap and demonstrate the inaccessibility of higher spatial frequencies when the larger GS gradient kernels are used. They also demonstrate that while radiality performs favourably to CoM estimation, MLE multi-emitter fitting is superior to radiality on a single frame basis.

To this point, all evaluations have been performed on a single frame, not including the resolution improvements to SRRF afforded by the temporal analysis. In order to investigate the effect of including the temporal information, emitter pairs were simulated with a 50% standard deviation in photon yield over 100 frames and analysed with each of the temporal radiality SRRF methods. Errors were calculated as the distance from the true emitter positions to the nearest local maxima in the single SRRF frame produced from 100 raw frames (Supplementary Fig. 4b). The results show a notable improvement in the precision over the most successful single frame radiality method and are comparable with MLE multi-emitter fitting. An example ground truth simulation (separation 275 nm) is shown in Supplementary Fig. 4c. A representative widefield image produced from an average projection of 100 frames shows that, at this separation, fluorophores would not be resolved using conventional imaging approaches (Supplementary Fig. 4c). Higher-order SRRF methods, TRA, TRPPM and TRAC4 produce images with progressively decreasing FWHM and increasing contrast allowing the two emitters to be better distinguished (Supplementary Fig. 4c). An MLE multi-emitter fitting reconstruction from the same 100 frame data set is shown for comparison (Supplementary Fig. 4c). Line profiles through the known positions of both emitters for the reconstructions in Supplementary Fig. 4c are shown in Supplementary Fig. 4d. Despite the complex inter-dependence of the radiality distributions these line profiles demonstrate that higher-order auto-cumulants do reduce the FWHM to some degree as well as de-noising and enhancing the SRRF reconstruction.

SRRF is not only applicable to low density datasets where one or two fluorophores appear close to one another. In order to quantitatively assess the performance of SRRF at higher densities of emitting fluorophores we have performed a comparative analysis across a wide range of parameters based on the analysis used by Geissbuehler et al[S12]. This analysis is based on a normalized visibility parameter that quantitatively assesses the extent to which two adjacent filamentous structures can be resolved. This analysis shows that at low rate ratios where the fluorophore on-time is significantly longer than the fluorophore off-time, resulting in high density datasets, SRRF consistently outperforms both the similar SOFI method and the benchmark localization method multi-emitter MLE (Supplementary Fig. 5). The visibility of the structures when analysed with SRRF was greater than both SOFI and MLE at low filament separations, low rate ratios, higher labelling densities, lower mean molecular signal and higher background.

To investigate experimentally the performance of SRRF, we acquired a low density super-resolution data set of Alexa Fluor 647 immuno-labelled fixed microtubules, fluorophore density was then estimated through analysis of the dataset using maximum likelihood estimation. A sequence of data sets with increasing fluorophore density was then generated by averaging frames from the low density data. This approach allows us to maintain an accurate fluorophore density and ground truth estimate,

which would not be possible with experimental high density datasets. Through the analysis of this data, the performance of SRRF was investigated, showing it is possible to resolve two approaching filaments even at ultra-high density (Supplementary Fig. 6, Methods). The averaging of low density data allowed the creation of datasets of length 990 and 198 frames with mean fluorophore separations of 74 nm and 31 nm respectively. Two microtubules were identified in the low density MLE dataset with a peak-to-peak separation of 74.7 nm. The mean localization precision of the 44454 localized fluorophores in the low density data was 15.9 nm giving a conservative estimate that the maximum possible separation of the filaments as 90 nm apart. A plot profile of the of the SRRF reconstruction at low density indicates the same 74.7 nm peak-to-peak separation while the FWHMs of the two filaments were 57.1 nm and 55.9 nm approximately equal to the 53 nm width expected for secondary antibody immuno-labelling of microtubules. While the visibility of the microtubules was reduced in the high density datasets the two microtubules were still resolved well (Supplementary Fig. 6).

Supplementary Note 3: Comparisons of SRRF performance against published super-resolution algorithms at different densities and for live-cell data

In order to qualify the advantages of SRRF, the three datasets presented in Fig. 2b were also analysed for six other super-resolution algorithms: three image-based approaches (3B[S13], SOFI[S7] and DeconSTORM[S14]) and three localization methods (DAOSTORM[S15], FALCON[S16] and ThunderSTORM[S3]). The corresponding single frame TIRF image for this region was also deconvolved using commercial deconvolution software (Supplementary Fig. 8a). Parameters for performing reconstruction using deconvolution and other super-resolution algorithms were determined from direct measurements on the raw data where appropriate (for example, image background estimations). Where other user-defined parameters were required, parameter sweeps were performed and the image most closely resembling the low density reconstruction from ThunderSTORM was chosen. The reconstructed images are shown in Supplementary Fig. 8c along with the computational time required to generate each displayed image. 3B analysis was not performed on the low density dataset as this dataset had tenfold more frames than the high density dataset and even when using cloud computing[S17] was expected to take approximately 20 days to run.

Of all the super-resolution algorithms tested, only SRRF and DeconSTORM were capable of producing images representative of the underlying structure at all three densities. However, the amount of time required for DeconSTORM computation was significantly longer than required for SRRF analysis (not including the time required to perform parameter sweeps in DeconSTORM) and DeconSTORM analysis is restricted to using square datasets. In this regard, SRRF is a considerably more efficient and flexible algorithm for performing super resolution analysis of datasets at a range of densities.

We also sought to quantify the similarity between the various reconstructed images, as it is clear from Supplementary Fig. 8c that many of the algorithms lose image information, especially at the higher densities. To achieve this, a line profile was taken across each image (location of profile shown in Supplementary Fig. 9a-b), and for each image this profile was averaged over 100 nm in the y direction to prevent bias from individual bright or mislocalized pixels. These profiles and the corresponding TIRF profile are plotted in Supplementary Figs. 9c-h. Supplementary Figs. 9c and d show the line profiles for the ultra-high density data reconstructions for SRRF compared with image-based methods and localization techniques respectively. As can be seen in Supplementary Fig. 9c, both SRRF and DeconSTORM both recovered all of the peaks visible in the TIRF profile and decreased their widths; DeconSTORM also succeeded in resolving a second peak within the large TIRF peak extending from 3500-4500 nm. The SOFI profile indicates blurring together of TIRF peaks (such as in the regions at 500-1500 nm and 2500-3500 nm), and 3B profile only recovers three of the peaks visible in the TIRF profile. The localization techniques also fail to recover the majority of peaks visible in the TIRF profile for ultra-high density data (Supplementary Fig. 9d). SRRF, SOFI and DeconSTORM displayed good fidelity in recovering peaks from high density data (Supplementary Fig. 9e), with all three algorithms now resolving two distinct peaks within the broad TIRF peak located at 3500-4500 nm. Again, 3B was incapable of recovering all of the features in the TIRF profile. At this density, the profiles for the localization techniques (Supplementary Fig. 9f) still lacked many of the features observable in the TIRF profile although more peaks were recovered than for the ultra-high density data (for example at ~750 nm and ~3200 nm). For the low density data, all of the image-based (Supplementary Fig. 9g) and localization (Supplementary Fig. 9h) methods recovered all of the peaks in the TIRF profiles and super-resolved multiple peaks within the single broad TIRF peaks at 1500-2500 nm (with the exception of SOFI) and 3500-4500 nm. All algorithms apart from SOFI also reconstructed sharper peaks than seen in the TIRF profile.

To fully assess the content of these measured line profiles, correlation analysis was performed for each line profile against a reference profile. The DAOSTORM low density profile was chosen as this

reference due to the previously reported good performance of DAOSTORM for both high and low density data[S18]. The Pearson's correlation coefficients between each profile from Supplementary Figs. 9b-d and the DAOSTORM low density profile are presented in Supplementary Fig. 10, where higher scores indicate better correlation. For the low density reconstructions SRRF had the second highest correlations score of all the tested algorithms, second only to FALCON and higher than that for the other localization algorithm tested, ThunderSTORM. For both the high and ultra-high density reconstructions SRRF had the highest correlation scores against the reference. Of particular note, SRRF reconstructions possessed similar correlation scores indicating that SRRF analysis is robust to different density datasets.

The image-based methods 3B, SOFI and DeconSTORM were also used to reconstruct a region of the anti-CD3&CD28 stimulated T-cell dataset shown in Fig. 4b and Supplementary Movie 8 (Supplementary Fig. 12). The deconvolved widefield image is also shown for comparison. SRRF analysis produced a reconstructed image containing all of the structural information within the deconvolved image with higher fidelity and improved contrast. Insufficient fluctuations in the acquired GFP data prevented SOFI from resolving any additional structure, and the DeconSTORM image contains typical ringing artefacts. While 3B succeeded in resolving part of the cell periphery, very little structure was recovered from the cell interior. Therefore for live-cell GFP data containing minimal intensity fluctuations SRRF performs similarly to deconvolution and produces images superior to those reconstructed with any other image-based super-resolution algorithm.

Supplementary Methods

Comparison with other super resolution algorithms For the comparisons with other super resolution algorithms in Supplementary Figs. 8-10, a 64x64 pixel region of interest was selected from each dataset (as acquired for Fig. 2). *3B:* 3B analysis was performed using Amazon Elastic Cloud Compute according to the method described in Hu et al[S17]. 81 overlapping 10x10 pixel masks were used on each image. For each image analysed, 20 c1.medium (compute optimized) instances were launched and monitored until the average number of iterations per mask exceeded 50 (as output by the scripts provided in Hu et al; this corresponds to 200 iterations as described in the original 3B paper[S13]). The average number of iterations per mask for was 62.9 for the ultra-high density dataset and 56.5 for the high density dataset. Quoted run times correspond to the total time required to analyse all 81 masks. A particles table was exported for the 3B results and the displayed images were rendered using normalized Gaussians of uncertainty 20 nm. *SOFI:* SOFI was run for orders 1-6 along with (fourth order) bSOFI[S19] for each density and the best image selected in each case. The ultra-high density reconstruction was produced using bSOFI with FWHM determined by the algorithm, and the high and low density reconstructions were produced using third order SOFI with a fixed FWHM. Run times indicate the total time to run all 6 orders and bSOFI. Analysis was performed using MATLAB running on a 2014 Intel Xeon E5-2687W v2 desktop processor. *DeconSTORM:* For DeconSTORM analysis the average background per pixel per frame was measured for each dataset and input into the algorithm, along with the measured sigma. For all reconstructions the gain parameter was set to 256 and 1000 iterations were used. Parameter sweeps were performed for the emitter probabilities α and β in the ranges α=[0.2, 0.99] and $\beta=[10^{-6}, 10^{-1}]$ and the best images selected. For the ultra-high and high density reconstructions, $\alpha=0.99$ and $\beta=10^{-6}$. For the low density reconstruction, $\alpha=0.5$ and $\beta=10^{-2}$. Analysis was performed using MATLAB running on a 2014 Intel Xeon E5-2687W v2 desktop processor. *DAOSTORM:* The PSF used for DAOSTORM analysis was generated from a frame in the low density dataset with PSF parameters as measured from this image. All other parameters in DAOSTORM were set as measured from the individual datasets. The resulting DAOSTORM particles tables were then rendered using normalized Gaussians of uncertainty 20 nm. Analysis was performed using a 2013 MacBook Pro. *FALCON:* FALCON was run using parameters as measured from the images and final images were rendered using normalised Gaussians of uncertainty 20 nm. Analysis was performed using MATLAB running on a 2013 Intel i7-4700MQ notebook processor with integrated graphics card. *ThunderSTORM:* ThunderSTORM analysis on each dataset was performed using the PSF: Integrated Gaussian method with maximum likelihood estimation and multi-emitter fitting analysis. Reconstructed images were rendered with normalized Gaussians of uncertainty measured from the molecular intensities. Analysis was run on a 2014 Intel Xeon E5-2687W v2 desktop processor. *Deconvolution:* Huygens deconvolution software was used to deconvolve the TIRF image using an estimated PSF based on the microscope parameters.

For the comparisons between different algorithms for the LifeAct-GFP T-cell image (Supplementary Fig. 12), algorithms were run as above. The SOFI reconstruction displayed is for third order SOFI with FWHM determined by the algorithm. The deconSTORM reconstruction displayed was created with $\alpha=0.99$ and $\beta=10^{-6}$ and 50 iterations.

Supplementary References

S

1. Burnette, D. T., Sengupta, P., Dai, Y., Lippincott-Schwartz, J. & Kachar, B. Bleaching/blinking assisted localization microscopy for superresolution imaging using standard fluorescent molecules. *Proc. Natl. Acad. Sci.* **108,** 21081–21086 (2011).

2. Ma, H., Long, F., Zeng, S. & Huang, Z.-L. Fast and precise algorithm based on maximum radial symmetry for single molecule localization. *Opt. Lett.* **37,** 2481–3 (2012).

3. Ovesný, M., Křížek, P., Borkovec, J., Svindrych, Z. & Hagen, G. M. ThunderSTORM: a comprehensive ImageJ plug-in for PALM and STORM data analysis and super-resolution imaging. *Bioinformatics* (2014).

4. Wang, Y. *et al.* Localization events-based sample drift correction for localization microscopy with redundant cross-correlation algorithm. *Opt. Express* **22,** 15982–91 (2014).

5. Huang, B., Wang, W., Bates, M. & Zhuang, X. Three-dimensional super-resolution imaging by stochastic optical reconstruction microscopy. *Science* **319,** 810–3 (2008).

6. Catmull, E. & Rom, R. in *Computer Aided Geometric Design* 317–326 (Elsevier, 1974).

7. Dertinger, T., Colyer, R., Iyer, G., Weiss, S. & Enderlein, J. Fast, background-free, 3D super-resolution optical fluctuation imaging (SOFI). *Proc. Natl. Acad. Sci. U. S. A.* **106,** 22287–92 (2009).

8. Mendel, J. M. Tutorial on higher-order statistics (spectra) in signal processing and system theory: theoretical results and some applications. *Proc. IEEE* **79,** 278–305 (1991).

9. Wahl, M., Gregor, I., Patting, M. & Enderlein, J. Fast calculation of fluorescence correlation data with asynchronous time-correlated single-photon counting. *Opt. Express* **11,** 3583–3591 (2003).

10. Rieger, B. & Stallinga, S. The lateral and axial localization uncertainty in super-resolution light microscopy. *ChemPhysChem* **15,** 664–670 (2014).

11. Henriques, R. *et al.* QuickPALM: 3D real-time photoactivation nanoscopy image processing in ImageJ. *Nature methods* **7,** 339–340 (2010).

12. Geissbuehler, S., Dellagiacoma, C. & Lasser, T. Comparison between SOFI and STORM. *Biomed. Opt. Express* **2,** 408–20 (2011).

13. Cox, S. *et al.* Bayesian localization microscopy reveals nanoscale podosome dynamics. *Nat. Methods* **9,** 195–200 (2011).

14. Mukamel, E. A., Babcock, H. & Zhuang, X. Statistical deconvolution for superresolution fluorescence microscopy. *Biophys. J.* **102,** 2391–400 (2012).

15. Holden, S. J., Uphoff, S. & Kapanidis, A. N. DAOSTORM: an algorithm for high- density super-resolution microscopy. *Nat. Methods* **8,** 279–80 (2011).

16. Min, J. *et al.* FALCON: fast and unbiased reconstruction of high-density super-resolution microscopy data. *Sci. Rep.* **4,** 4577 (2014).

17. Hu, Y. S., Nan, X., Sengupta, P., Lippincott-Schwartz, J. & Cang, H. Accelerating 3B single-molecule super-resolution microscopy with cloud computing. *Nat. Methods* **10,** 96–7 (2013).

18. Sage, D. *et al.* Quantitative evaluation of software packages for single-molecule localization microscopy. *Nat. Methods* 1–12 (2015). doi:10.1038/nmeth.3442

19. Geissbuehler, S. *et al.* Mapping molecular statistics with balanced super-resolution optical fluctuation imaging (bSOFI). *Opt. Nanoscopy* **1,** 4 (2012).
