# Supplementary material for: Fast live-cell conventional fluorophore nanoscopy with ImageJ through super-resolution radial fluctuations
Source: Nat Commun. 2016 Aug 12;7:12471. doi: 10.1038/ncomms12471 (PMC4990649; doi:10.1038/ncomms12471)
Supplement: Supplementary Software — The SRRF algorithm. The SRRF algorithm source code and plugin for ImageJ and Fiji are provided along with a manual providing instructions for installation and use. [file ncomms12471-s10.zip › NanoJ-SRRF supplementary software/NanoJ User Manual Version 1.1R0.pdf]

NanoJ User Manual

Table of Contents

Introduction 2

Installing NanoJ into Fiji 2

NanoJ file format – .nji 4

SRRF Analysis dialog 5

Ring Radius (1) 5
Radiality Magnification (2) 6
Axes in Ring (3) 6
Show Advanced Settings (4) 6
Temporal Radiality Maximum (TRM) (4.1) 7
Temporal Radiality Average (TRA) (4.2) 7
Temporal Radiality Pairwise Product Mean (TRPPM) (4.3) 7
Temporal Radiality Auto-Correlations (TRAC) (4.4) 7
Temporal Subtraction (4.5) 8
Remove Positivity Constraint (4.6) 8
Renormalize (4.7) 9
Do Gradient Smoothing (4.8) 9
Do Intensity Weighting (4.9) 9
Do Gradient Weighting (4.10) 9
Time-lapse SRRF Reconstructions (5) 10
Cropping Data (6) 11
Drift Correction (7) 11
Do Batch Analysis (8) 13
Do Preview (9) 13

Logging SRRF settings 13

Known issues 13

Aparapi crashes 13
'WARNING: Aborting NanoJ Execution' 14
SRRF settings are not updating 14
Black borders surrounding images 14

Testing data 15

Introduction

NanoJ is a Fiji plugin which allows for reconstruction of super resolution images from PALM/STORM and widefield data sets through SRRF analysis. This guide describes how to set up and run NanoJ and how to best select settings for the type of data being analysed.
This manual was produced using NanoJ release version 1.1Stable0 running in Fiji with ImageJ 1.50e. Testing was done on both Mac and Windows (both 64 bit) with Java version 1.6.

Installing NanoJ into Fiji

1. Open Fiji, go to the ‘Help’ menu and then ‘Update Fiji’.
2. Once the checksummer has run, the window shown below should appear (this window may contain items depending on the current status of your version of Fiji – this won’t affect NanoJ installation). Press ‘Manage update sites’.

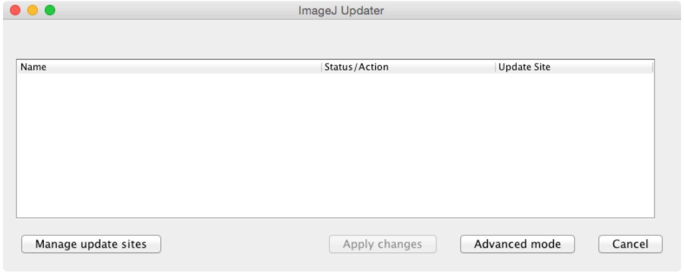

3. Press ‘Add my site’.

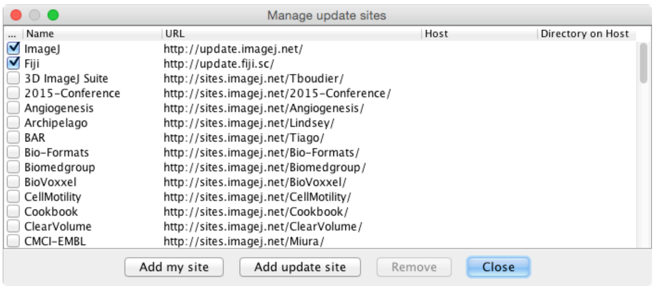

4. In the ‘ImageJ Wiki Account’ field in the next window, enter ‘NanoJ’. Repeat this again for the ImageJ Wiki account ‘NanoJ-SRRF’.

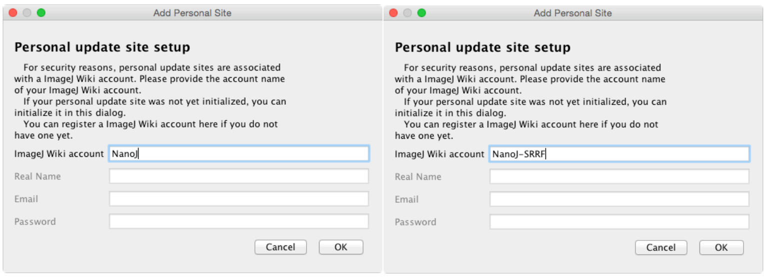

5. The list of update sites should now look like the following:

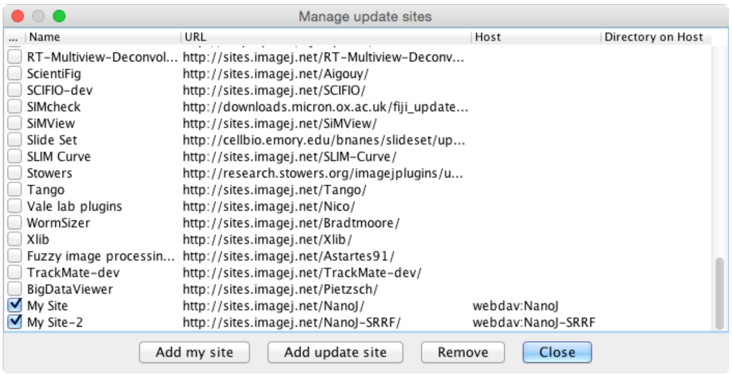

For future reference it is useful to rename 'My Site' and 'My Site-2' to 'NanoJ' and 'NanoJ-SRRF' – this can be done by just clicking on 'My Site' and typing.

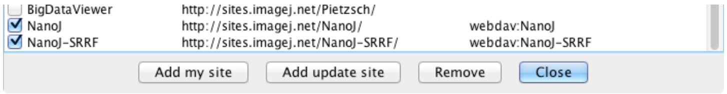

6. Close the 'Manage update sites' window and apply changes when prompted.

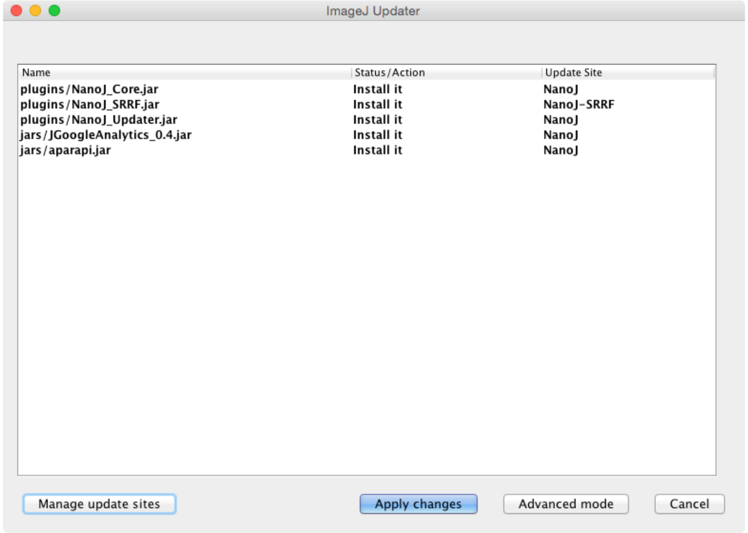

7. If successful you should get the following message:

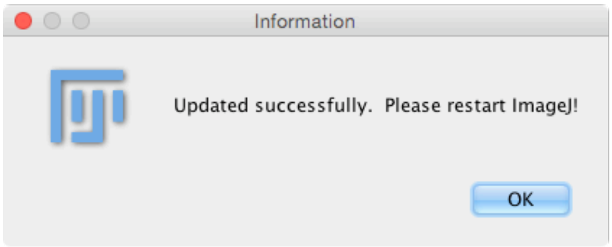

8. Upon restarting Fiji, you should now have the following options in the Plugins menu:

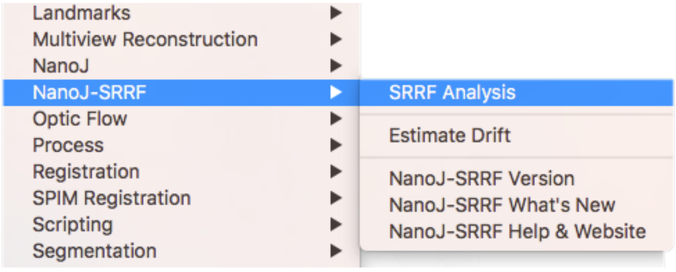

Congratulations! You are now ready to start using NanoJ!

NanoJ file format – .nji

NanoJ allows you to save large image sequences as a series of .nji files which loaded back into NanoJ quickly and easily at a later date. For example, the following raw data set can be saved as a series of .nji files by selecting the 'Save Image as NJI…' option from the NanoJ menu. This conversion should work on any file type imported into Fiji.

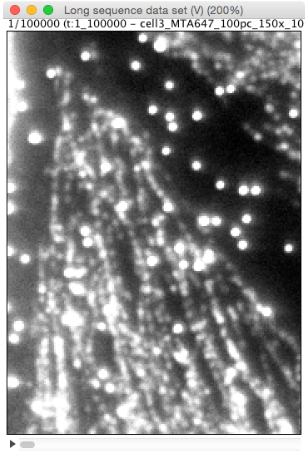

The window below shows the original .tif data set (8.55GB) and the six .nji files created by NanoJ (total size c.a. 6.2GB).

Name	Date Modified	Size	Kind
Long sequence data set.tif	Today 20:15	8.55 GB	TIFF ir
Long sequence data set-000.nji	Today 20:34	1.1 GB	Unix E
Long sequence data set-001.nji	Today 20:37	1.1 GB	Unix E
Long sequence data set-002.nji	Today 20:39	1.1 GB	Unix E
Long sequence data set-003.nji	Today 20:41	1.1 GB	Unix E
Long sequence data set-004.nji	Today 20:44	1.1 GB	Unix E
Long sequence data set-005.nji	Today 20:45	726.4 MB	Unix E

.nji files can be opened from File – Import – NanoJ file or from the NanoJ menu, and load into Fiji faster than the original large data set. The whole data set is opened by selecting any one of the .nji files created; you do not need to open each .nji file separately.

SRRF Analysis dialog

To run SRRF analysis on a data set, select ‘SRRF Analysis’ from the NanoJ-SRRF menu. If you do not currently have an active image stack open in Fiji then you will be prompted to load one before analysis commences. The default ‘Super-Resolution Radial Fluctuations…’ dialog box which will open the first time you use NanoJ will look as below.

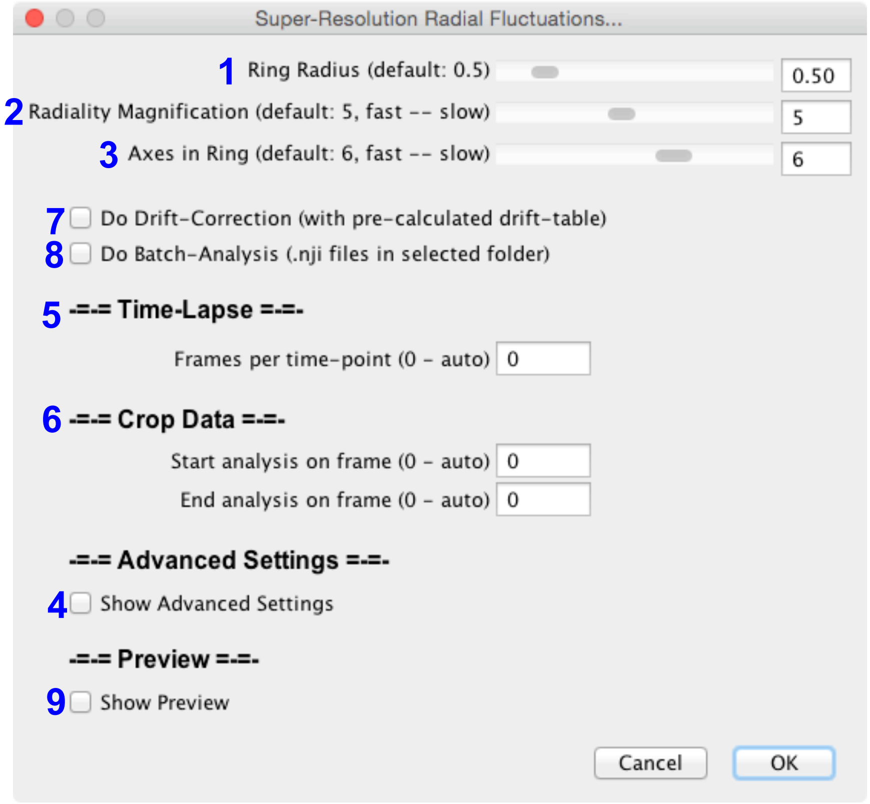

Note: on SRRF 1.0Stable4 a new option “Max temporal analysis block size” has been added. This value modulates the way the data is managed internally, if SRRF is crashing during analysis try a smaller value (e.g.: 100-500), it will make SRRF more stable at a small cost of speed and reconstruction quality.

Ring Radius (1)

To calculate the radiality at each co-ordinate in the image, SRRF needs to calculate the intensity gradients in a ring surrounding each co-ordinate. The ring radius setting determines the radius of this ring. For low density data sets, setting this radius to be low will increase resolution of the image; however, setting the radius too low in noisy datasets will reduce precision and may introduce patterning into the resulting image.

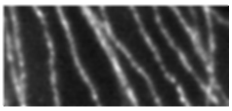
Ring radius = 0.1

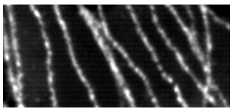
Ring radius = 0.5

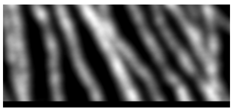
Ring radius = 3

Radiality Magnification (2)

Radiality is calculated on a magnified pixel grid; the radiality magnification setting determines how many magnified pixels each original image pixel is split into. For example, a radiality magnification of 5 will split each image pixel into 5x5 magnified pixels. The three images below show an example of changing the radiality magnification; the cost of increasing magnification is increased computational time.

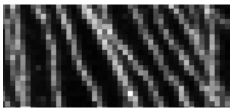

Magnification = 1
(44 x 20 pixels)

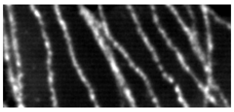

Magnification = 5
(220 x 100 pixels)

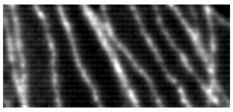

Magnification = 10
(440 x 200 pixels)

Axes in Ring (3)

As described for the ring radius, radiality is calculated in a ring surrounding a given co-ordinate (magnified pixel). The axes in ring setting determines the number of points around the circumference of this ring that are used in the calculation. Use of a higher number of symmetry axes increases fidelity but increases computational time. The number of axes should only be reduced if computational time needs to be greatly reduced e.g. for preliminary checks of data.

Show Advanced Settings (4)

Ticking this checkbox will bring up the 'SRRF Advanced Settings…' dialog box as shown below:

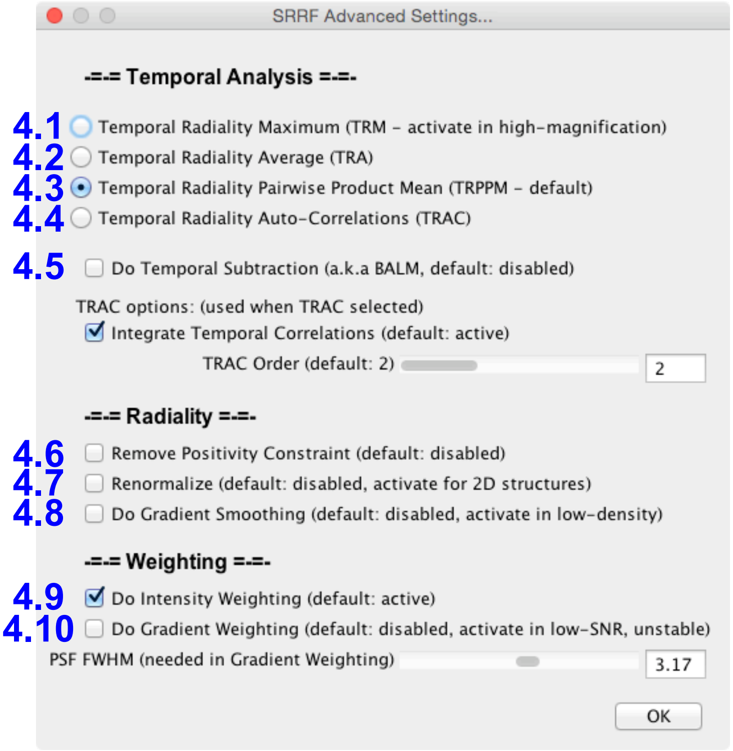

This dialog box is split into three main sections: Temporal Analysis, which determines the method by which the final image is combined following the radiality transform calculation (Table 1); Radiality, which are settings relating to the calculating the radiality transform (Table 2); and Weighting (Table 2).

Temporal Radiality Maximum (TRM) (4.1)

TRM displays the final image as a maximum intensity projection of the radiality transforms of each individual frame in the original image. TRM works most successfully for sparse data sets i.e. classical PALM/STORM experiments.

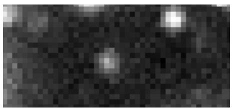

Individual frame from sparse data set

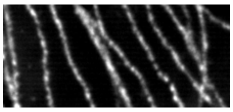

TRM reconstruction (all other parameters set at default)

Temporal Radiality Average (TRA) (4.2)

TRA displays the final image as an average intensity projection of the radiality transforms of each individual frame in the original image. TRA is often a good starting point for analysis, especially for blinking data sets where fluorophores overlap or data sets acquired where fluorophores display limited intensity fluctuations such as in conventional TIRF or widefield microscopy.

Temporal Radiality Pairwise Product Mean (TRPPM) (4.3)

TRPPM displays the final image as the raw second moment (related to the variance) of the radiality integrated over the time series. Images produced with TRPPM contain a similar level of detail and resolution as TRA, but can have additional noise suppression.

Temporal Radiality Auto-Correlations (TRAC) (4.4)

TRAC performs auto-correlation analysis similar to that used in the SOFI super resolution technique published elsewhere. TRAC works successfully for dense data sets with overlapping fluorophores.

The ‘Integrate Temporal Correlations’ checkbox should be ticked in all cases where an individual fluorophore can reappear at later times in the acquisition. An example of when it may be beneficial to uncheck this box is a PALM data set where the control of fluorophore activation and bleaching ensures that the same fluorophore does not appear in more than one burst.

The TRAC order slider changes the order of auto-cumulant analysis performed. Higher TRAC orders should provide higher resolution, but will also result in a large dynamic range where the contrast may be challenging to adjust.

Temporal Subtraction (4.5)

Temporal subtraction diminishes the impact of fluorophores which are in an emitting state for more than one frame by subtracting intensity between adjacent frames. This is most useful for data sets where there is fluorophore blinking (i.e. not a GFP or widefield data set) with overlapping fluorophores.

Table 1 – Different temporal analyses for non-sparse data sets

	Mid-density blinking data set	Non-blinking data set
Raw frame from data	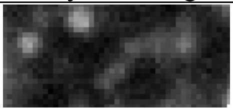	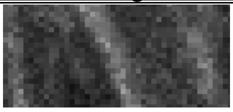
Average intensity projection	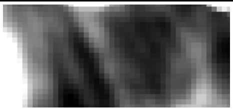	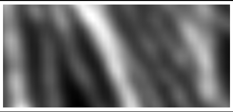
TRM	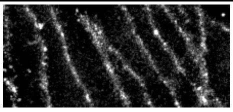	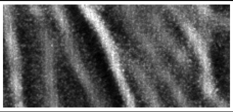
TRA	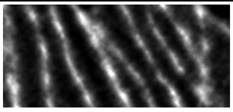 *	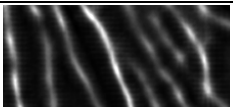
TRPPM	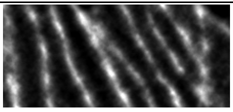 *	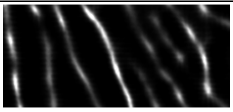
TRAC2	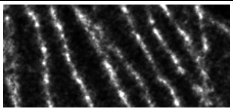 *	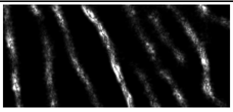
TRAC3	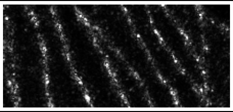 *	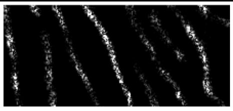
TRAC4	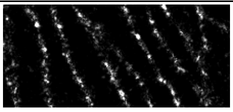 *	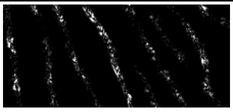
* indicates that temporal subtraction was also applied		

Remove Positivity Constraint (4.6)

In the radiality calculations, it is possible to get negative values which correspond to a positive probability that there is no fluorophore at a given location. In cases where there is a possibility of a fluorophore emitting at a later time the radiality should always be constrained to positive values to avoid the absence of a fluorophore in a single frame affecting the positive radiality in subsequent frames. However, this option can be selected in order to increase contrast in single frame Radiality analyses where temporal statistics are not applied.

Renormalize (4.7)

SRRF has a tendency to enhance edges in images if the renormalize option is not selected. Therefore if non-filamentous structures or regions of homogenous fluorescence are being analysed the renormalize option should be selected.

Do Gradient Smoothing (4.8)

Ticking the gradient smoothing checkbox increases the size of the kernel used for calculating the radiality and results in a smoothing effect on the image. The downside of this is that inclusion of data from a larger number of pixels can result in bulges in structures and a decrease in spatial resolution.

Do Intensity Weighting (4.9)

The spatial component of SRRF analysis, the radiality map, contains many small radiality peaks in the noise. While these are suppressed if subsequent temporal analysis is performed (i.e. TRPPM or TRAC), they are not if TRM or TRA is used to display the image. Therefore, to denoise TRM/TRA images intensity weighting is applied to enhance radiality peaks arising from high-intensity regions. For TRPPM/TRAC analysis intensity weighting is less critical.

Do Gradient Weighting (4.10)

Gradient weighting denoises images by increasing the contrast of point-like objects and is thus useful in data sets where blinking can be observed. It is less useful in widefield-type data sets. The PSF width used for gradient weighting does not have to be exact but should be a good approximation. Setting the width too low can cause patterning, while too high will broaden structures in the image.

Table 2 - Advanced radiality weighting and smoothing options for different density data sets

	Sparse data	Mid-density data	Widefield data
Default weighting/ smoothing	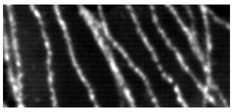	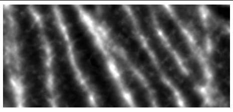	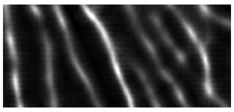
Disable Positivity Constraint	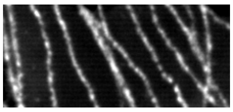	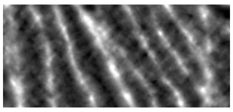	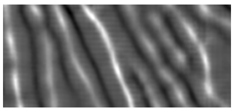
Re-normalize	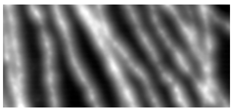	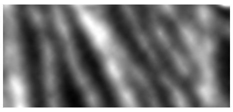	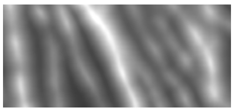
Gradient smoothing	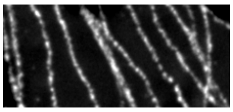	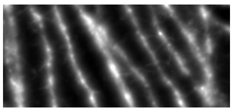	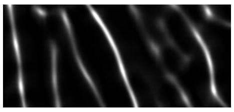
Untick intensity weighting	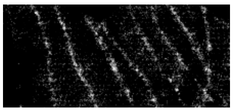	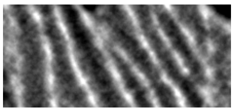	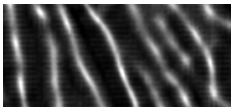
Enable gradient weighting	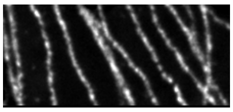	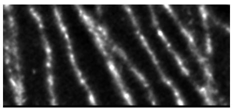	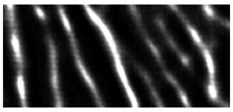

For each image, all other settings were the recommended defaults. Sparse data images were all made with TRM temporal analysis; Mid-density data images were all made with TRA temporal analysis with temporal subtraction; Widefield data images were all made with TRA temporal analysis.

Time-lapse SRRF Reconstructions (5)

For all the analysis described above, all frames in the raw image stack are used to create the final SRRF image (when this value is set to 0). However, for live-cell data sets where there are dynamic processes, SRRF time-lapse series can be produced.

The number of raw frames used to create each SRRF frame is set by the value in this box. For example, if this value is set to 100, each frame in the SRRF time-lapse will have been reconstructed from 100 raw data frames. If the exposure time on the frame was originally 10ms, then this would translate to an effective SRRF time-lapse rate of 1 frame per second (1Hz). Each group of frames is treated independently as an individual image stack – temporal analysis will only take into account frames within each group.

Cropping Data (6)

Occasionally there are frames at the beginning and/or end of a data set which should not be included in analysis. For example, saturated frames which are commonly present in early frames of acquisition will produce artefacts within reconstructed images. Frames at the end of acquisitions may also contain no signal as a result of bleaching and will not improve the final image.

By defining start and end frames in the GUI, unwanted frames will be cropped out of the data set and SRRF analysis will only be performed on the remaining subset of frames.

Drift Correction (7)

NanoJ features a fiducial marker-based drift correction system. By checking this box, a dialog window will open asking you to select the appropriate drift table to apply to the data set.

Prior to running SRRF analysis with drift correction, a drift table must be created for the data set. This is done by selecting 'Estimate Drift' from the NanoJ-SRRF menu. After choosing a location to save the drift table, the following dialog box will appear:

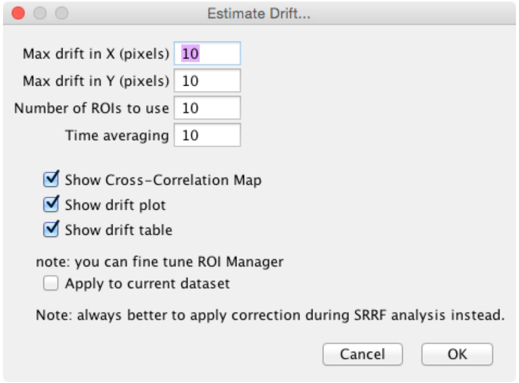

The ROI manager will also open with the current estimation of fiducial marker locations contained:

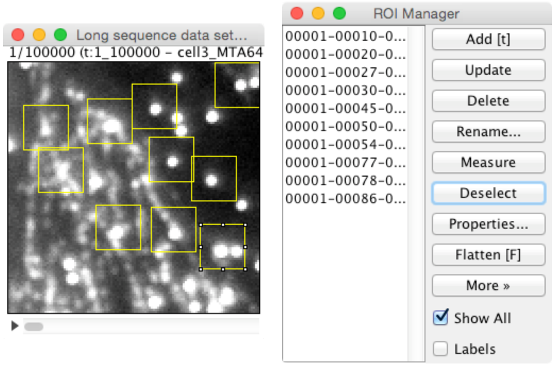

Firstly, scroll to the end of the data to check whether the size of the yellow drift ROIs are sufficient to contain the fiducial markers.

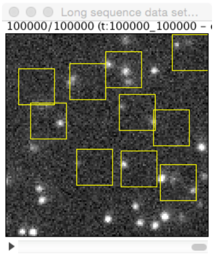

In the case of this dataset, the drift in the x direction is taking the fiducial markers just outside the boundaries of the ROIs. By adjusting the ‘Max drift in X’ and ‘Max drift in Y’ values the ROIs can be reshaped such that the extent of the drift is better described while always containing the fiducial markers.

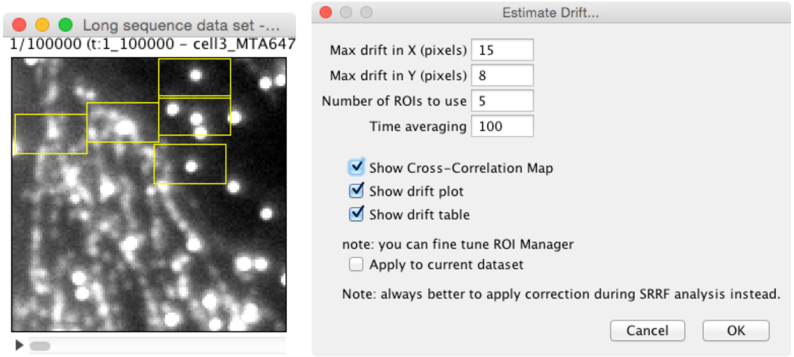

In the above example, the ‘Time averaging’ value has been set to 100: this is the approximate number of frames over which there is minimal drift for this data set and so can be averaged together. Finally, individual ROIs can be relocated by selecting them in the ROI manager and then dragging them to the desired locations:

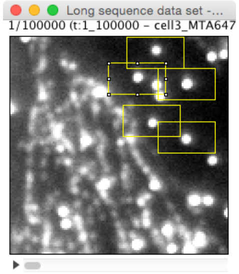

The three ‘Show…’ checkboxes do not affect the drift correction analysis itself or the saving of the drift table – these simply allow you to see the drift plots etc. once drift correction is completed. The ‘Apply to current dataset’ checkbox drift corrects the raw data set itself – if unchecked, a drift table will be generated which can be applied as SRRF analysis is run on the data. We recommended drift correction during SRRF analysis (i.e. leaving the last checkbox unchecked). Click ‘OK’ to run drift correction and generate the .xls drift table file.

This file can then be selected when the ‘Do Drift-Correction’ checkbox is checked during SRRF analysis.

Do Batch Analysis (8)

If you have a series of files saved in the .nji format, you can set NanoJ to run through the files and perform SRRF analysis on all of them. The same SRRF settings will be used for each analysis.

Do Preview (9)

Ticking this box will bring up a preview of SRRF analysis on the current frame of the data set. As this preview is generated on a single frame basis, it does not contain any improvements from temporal analysis, only the settings affecting spatial analysis (such as ring radius, radiality magnification and smoothing/weighting).

Logging SRRF settings

NanoJ reports information into the Fiji Log – this information is typically the settings selected by the user for SRRF analysis. This log can be saved by the user for referencing settings at a later date.

The remaining time left in the current SRRF analysis can be displayed in the Fiji message bar. If this is not appearing, then go to the NanoJ menu and then 'Debug Preferences'. This should bring up the following dialog box:

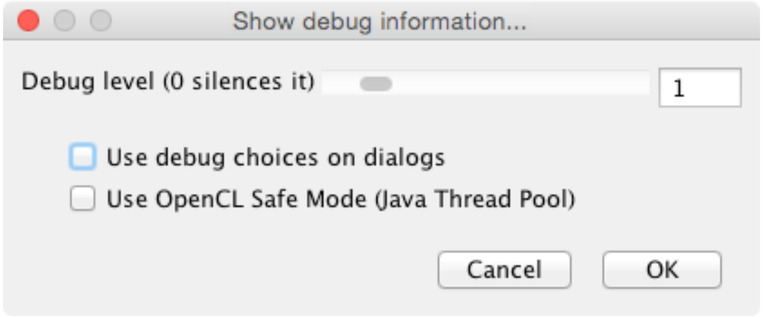

Set the debug level to 1 and then the remaining time should be displayed in the Fiji message bar for subsequent analysis (you may need to restart Fiji for this to take effect). Debug levels higher than 1 are for use by developers.

Known issues

Aparapi crashes

NanoJ uses the Aparapi API to parallelize computation – this allows for code to be executed on the GPU via OpenCL. Aparapi does have a slight tendency to crash for unknown reasons – if this happens then an error message will appear either in the Fiji console or log.

If Aparapi is crashing frequently then processing can be reverted to the CPU; while this is slower to perform calculations, it is much more stable. To force NanoJ to run on the CPU by default, open the ‘Show debug information…’ dialog (as described in ‘Logging SRRF settings’) and tick the ‘Use OpenCL Safe Mode (Java Thread Pool)’ box.

‘WARNING: Aborting NanoJ Execution’

Sometimes, when SRRF analysis is set to run, computation will not start and a warning message will appear in the Fiji message bar. The most common cause of this is a ‘blocking error’. NanoJ splits up images into xyt blocks to better handle large data sets, and very occasionally will not be able to find an appropriate block size (this can be seen in the log, where a message saying ‘NanoJ: Blocksize set to 0x0x0’ appears).

To get round this problem, the size of the image must be changed slightly. Block size calculations are most stable if the image is cropped to a square, but this is not mandatory and usually any crop will solve this problem.

NanoJ execution will also abort if the number of frames in a drift table does not match the number of frames in the image stack. If this happens then a dialog box should pop up with a message to this effect.

NanoJ execution can also be stopped manually (e.g. if SRRF analysis is started with the wrong settings) by selecting ‘Stop NanoJ Execution…’ from the NanoJ menu.

SRRF settings are not updating

Sometimes the settings in SRRF ‘stick’ and changing them appears to have no effect. If this happens then the Preferences can be reset by selecting ‘Reset Preferences…’ from the NanoJ menu. After selecting this option it is very important that you restart Fiji immediately (without doing any more image processing/analysis actions, opening files, saving images etc.) as it deletes the main Fiji preferences file. This will reset NanoJ settings to their default values.

Black borders surrounding images

A black border surrounding the reconstructed image can be seen if certain settings are selected. For example, if there is a large amount of drift then there will be a larger border surrounding the image. This is not a fault; it is simply a mechanism to avoid reconstructing images with incomplete information.

Testing data

For initial familiarization with the SRRF plugin, we recommend testing it out with data from the ISBI localization challenge, such as the high density tubulin dataset available for download at: http://bigwww.epfl.ch/smlm/datasets/index.html?p=real-hd.
